# One-Pot Synthesis of Abietane-Type Hydroxamic Acids: Process Optimization and Mechanistic Insights

**DOI:** 10.3390/molecules31101637

**Published:** 2026-05-13

**Authors:** William E. Mendoza-Hernández, Ramón J. Zaragozá, Urbano Díaz, Miguel A. González-Cardenete

**Affiliations:** 1 Instituto de Tecnología Química, Consejo Superior de Investigaciones Científicas-Universitat Politècnica de València, 46022 Valencia, Spain; wemenher@itq.upv.es (W.E.M.-H.); udiaz@itq.upv.es (U.D.); 2 Departamento de Química Orgánica, Universitat de Valencia, Dr. Moliner 50, 46100 Burjassot, Valencia, Spain; ramon.j.zaragoza@uv.es

**Keywords:** abietane, abietic acid, dehydroabietic acid, synthesis, hydroxamic acid

## Abstract

The synthesis of hydroxamic acids from sterically hindered substrates, such as abietane-type resin acids, remains a synthetic challenge due to the congestion of the tricyclic skeleton. This study reports an efficient one-pot protocol for the direct conversion of abietic and dehydroabietic acids into their corresponding hydroxamic derivatives, achieving 65% and 74% isolated yields, respectively. Systematic screening of activating agents identified diethyl chlorophosphate (DCP) as the reagent for the hydroxyamidation. A critical finding of this work is that the optimization of the isolation process specifically minimizing the water amount during aqueous work-up was key to recovering these polar products and preventing important yield loss. The reaction proceeds through diethyl phosphate mixed anhydride intermediate, which was successfully isolated, providing direct experimental evidence of the activation pathway. The reaction mechanism was further elucidated using Density Functional Theory (DFT) calculations at the M062X/6-31G** level, identifying a concerted transition state for the simultaneous addition of hydroxylamine and expulsion of the phosphate group. Furthermore, the study rationalizes the observed chemoselectivity; although the ester is the more stable thermodynamic product, the formation of the N-hydroxy amide is kinetically favored through a substantially lower activation barrier. This combined experimental and theoretical approach establishes a practical and scalable methodology for the functionalization of abundant similar natural terpenoids.

## 1. Introduction

**Figure 1:**
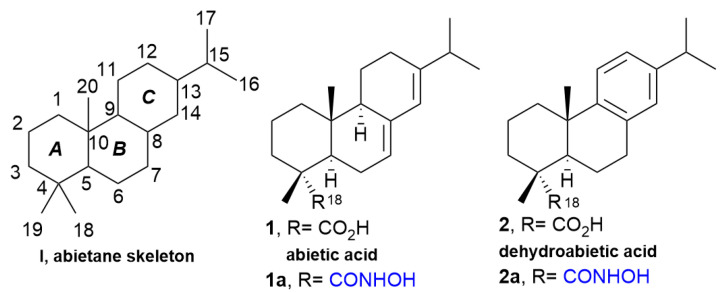
Structures of starting resin acids (**1**, **2**) and their corresponding hydroxamic acid derivatives (**1a**, **2a**).

Abietane-type diterpenoids, such as abietic acid (**1**, Figure 1) and dehydroabietic acid (**2**, Figure 1), represent a class of tricyclic natural products characterized by wide structural diversity and occurrence in coniferous resins [1,2]. These compounds have long served as versatile chiral scaffolds in organic synthesis for the preparation of bioactive derivatives and the semisynthesis of other complex natural products [3,4]. However, the functionalization of the C18 carboxyl group in the abietane skeleton remains a formidable synthetic challenge. This difficulty arises from the severe steric hindrance imposed by the rigid tricyclic system and the proximity of the methyl groups at the C4 and C10 positions [5].

Hydroxamic acids, or N-hydroxy amides, are of particular interest in organic and medicinal chemistry due to their unique ability to form stable chelates with transition metal ions and their prevalence in enzyme inhibitors [6,7]. While the incorporation of a hydroxamic acid moiety has been shown to enhance the properties of various terpenoids, including derivatives of betulin, oleanolic acid, and glycyrrhetinic acid [8,9,10], reports on abietane-derived hydroxamates are remarkably scarce. A landmark study by Bardyshev reported the preparation of abietohydroxamic acid (**1a**, Figure 1) in only 14% yield through the formation of intermediate anhydrides [11]. Despite this early precedent, the method lacked optimization for highly hindered substrates, and a detailed understanding of the factors governing the competitive N- vs. O-attack of hydroxylamine on such congested centers was not established.

Traditional methods for hydroxamic acid synthesis often involve the reaction of esters or acid chlorides with hydroxylamine [12,13]. Nevertheless, these standard procedures failed in our hands to derivatize either abietic acid or dehydroabietic acid. In this context, the development of stepwise activation strategies is common to channel reactivity in a controlled manner. Diethyl chlorophosphate (DCP) has emerged as a reagent for the in situ generation of reactive mixed anhydride intermediates, yet its application to the synthesis of hindered abietane hydroxamates has not been systematically explored until now.

In this work, we describe an optimized one-pot protocol for the conversion of abietic and dehydroabietic acids into their corresponding hydroxamic derivatives using DCP as the activating agent. Beyond the synthetic optimization, we provide the first comprehensive mechanistic rationalization of the observed chemoselectivity through Density Functional Theory (DFT) calculations. This work expands upon our preliminary findings reported at the 29th International Electronic Conference on Synthetic Organic Chemistry [14]. By establishing a unified energy reference point, we demonstrate how the reaction is governed by strict kinetic control. This control favors the formation of the N-hydroxy amide over the amino ester, which is the thermodynamically more stable product, successfully overcoming the steric demand of the diterpene skeleton.

## 2. Results and Discussion

### 2.1. Screening of Activation Methods and Mechanistic Discovery

**Scheme 1:**
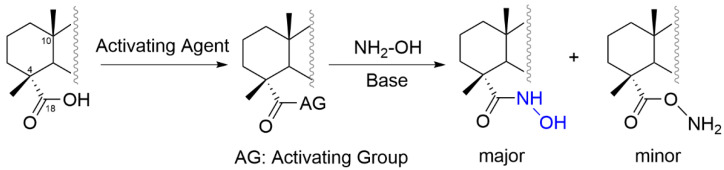
General relay-activation strategy for the synthesis of hindered abietane hydroxamic acids.

The synthesis of hydroxamic acids derived from abietane-type resin acids represents a formidable synthetic challenge that has long hindered the functionalization of these natural products. This difficulty arises primarily from the steric hindrance surrounding the C18 carboxyl group. This specific carbon center is effectively shielded by the rigid tricyclic framework of the diterpene skeleton and is further congested by the presence of axial methyl groups located at the C4 and C10 positions. Such a dense chemical environment precludes the possibility of direct functionalization of the carboxylic acid, thereby necessitating an indirect activation pathway via a relay-activation mechanism to overcome the high organizational and steric barriers of the system. This general activation approach, designed to bypass the steric congestion of the resin acid, is illustrated in Scheme 1.

Attempts to facilitate this transformation using ethyl chloroformate and triethylamine to form a classic mixed anhydride resulted in the complete recovery of the starting material. We subsequently studied a more aggressive activation via the acyl chloride using thionyl chloride (SOCl_2_) and pyridine. However, the used conditions favored the formation of the symmetric anhydride of dehydroabietic acid over the desired attack by the nitrogen nucleophile. Furthermore, modern coupling reagents such as EDC/DMAP were evaluated, but these yielded highly complex mixtures with low selectivity, making the purification of the target hydroxamic acid practically impossible.

We systematically evaluated widely used carboxyl activation strategies to benchmark the reactivity of the C18 center, before arriving to an optimized phosphorus-mediated route. We initially explored the use of propylphosphonic anhydride (PPAA) in acetonitrile at room temperature [15]. For abietic acid (**1**), this reagent resulted in the complete recovery of the starting material (Table 1, Entry 1). Interestingly, when PPAA was applied to dehydroabietic acid (**2**), the results were inconsistent; specific experiments yielded the desired hydroxamic acid with a modest yield of approximately 20%, but the reactions were characterized by poor selectivity and the formation of unknown side products (Table 1, Entry 2).

**Scheme 2:**
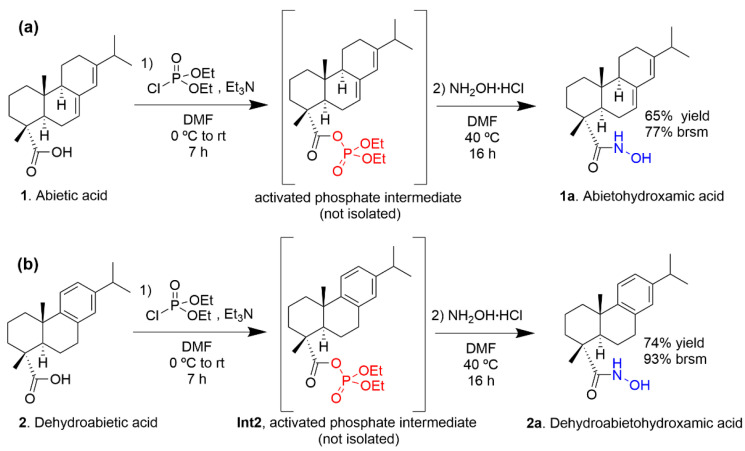
Optimized one-pot synthesis of abietohydroxamic acid (**1a**) and dehydroabietohydroxamic acid (**2a**) via an in situ generated diethyl phosphate mixed anhydride intermediate. Brsm: based on recovered starting material.

Following these trials, we explored the use of diethyl phosphorocyanidate (DEPC) [16]. When the reaction was performed by mixing all reagents simultaneously with substrate **1**, we observed a low yield of 13.7% (Table 1, Entry 3). However, these results were fundamental in unraveling the reaction mechanism. By employing dehydroabietic acid (**2**), we successfully isolated and characterized the corresponding stable diethyl phosphate mixed anhydride intermediate (**Int2**, Scheme 2b) (Table 1, Entry 4). The subsequent transformation of this isolated intermediate into the desired hydroxamic acid **2a** was achieved in a separate step by reaction with hydroxylamine in DMF (Table 1, Entry 5).

**Table 1:** Optimization of the activation and hydroxyamidation conditions for abietic and dehydroabietic acids (**1** and **2**) (see also Scheme 2).

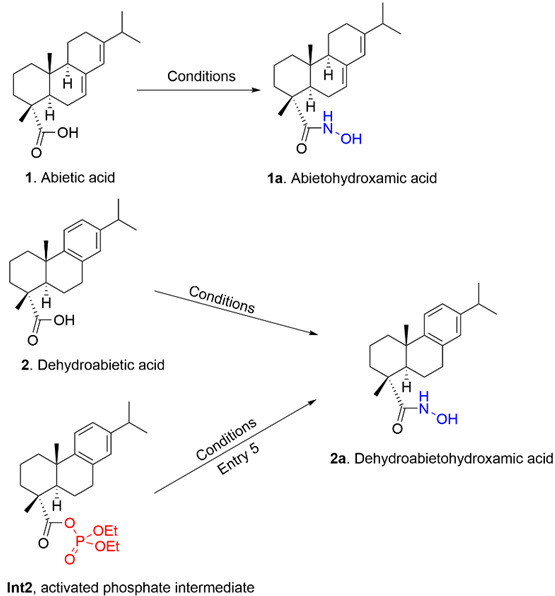					
Entry	SM ^2^	Reagent ^3^	Solvent	T °C	Results
1	1	PPAA, Et_3_N, NH_2_OH· HCl	MeCN	rt	SM recovered
2	2	PPAA, Et_3_N, NH_2_OH· HCl	MeCN	rt	20.1% yield of **2a**
3	1	DEPC, Et_3_N, NH_2_OH/Et_3_N	THF	rt-40 °C	13.7% yield of **1a**
4	2	DEPC, Et_3_N, NH_2_OH/Et_3_N	THF	rt	Phosphate intermediate isolated (**Int2**)
5	Int2	NH_2_OH· HCl, Et_3_N	DMF	rt	Confirmed formation of **2a**
6	1	(1) DCP, Et_3_N (2) NH_2_OH· HCl	DMF one pot	(1) 0–rt (2) 40 °C	17.3% yield of **1a**
7	2	(1) DCP, Et_3_N (2) NH_2_OH· HCl	DMF one pot	(1) 0–rt (2) rt	34.2% yield of **2a**
8 ^1^	1	(1) DCP, Et_3_N (2) NH_2_OH· HCl	DMF one pot	(1) 0–rt (2) 40 °C	64.6% yield of **1a**
9 ^1^	2	(1) DCP, Et_3_N (2) NH_2_OH· HCl	DMF one pot	(1) 0–rt (2) 40 °C	73.8% yield of **2a**

^1^ Optimized work-up utilizing a minimized aqueous volume for both the initial quenching and each subsequent washing step of the organic phase. ^2^ SM: starting material or limiting reactant. ^3^ PPAA: propylphosphonic anhydride; DEPC: diethyl phosphorocyanidate; DCP: diethyl chlorophosphate.

The outcomes of Entry 3 and Entry 4 provided the critical mechanistic validation needed to move forward. These results confirmed that phosphorus-mediated activation was the correct chemical strategy. Therefore, it was envisaged that phosphorylation with diethyl chlorophosphate (DCP) would operate via a nearly identical activation pathway, but as a more cost-effective phosphorous source. To conclude this screening phase, we transitioned to a one-pot protocol using DCP and DMF at 40 °C. While this confirmed the chemical viability of the method, the initial yields obtained using standard isolation procedures remained disappointing: 17% starting from abietic acid (**1**) and 34% from dehydroabietic acid (**2**) (Table 1, Entry 6 and Entry 7, respectively).

### 2.2. Optimization One-Pot Synthesis

The focus of the investigation then shifted to the optimization of the isolation process (work-up). Our findings revealed that the true bottleneck was not the chemical reactivity, but rather the loss of product during extraction. The resin acid derivatives studied here possess an amphiphilic nature, which causes an important portion of the hydroxamic acid to partition back into the aqueous phase during the traditional washing steps.

The impact of this optimization is most evident when comparing the physical state and purity of the crude products. In the initial trials using a standard volume of 20 mL of water (Table 1, Entries 6), the resulting crude was a dry yellow foam. However, ^1^H NMR analysis revealed a discouraging 1:1 ratio between the hydroxamic acid and the starting carboxylic acid, confirming a considerable product loss.

To address this, we implemented a modified work-up by drastically reducing the water volume to 5 mL. This change resulted in a crude product that appeared as a yellowish solution in residual DMF. Crucially, the ^1^H NMR purity improved to a 3.3:1 ratio (product to starting acid). This decision represented a strategic trade-off: we deliberately sacrificed the total removal of DMF in the crude stage to ensure the maximum recovery of hydroxamic acid. While residual DMF can sometimes complicate purification, we found that the remaining volume was sufficiently small to not interfere with the subsequent flash column chromatography fractions.

By applying this optimized lower water amount work-up, the isolated yields increased up to 64.6% for abietic acid (Table 1, Entry 8) and 73.8% for dehydroabietic acid (Table 1, Entry 9). The finalized optimized one-pot protocol is illustrated in Scheme 2.

**Figure 2:**
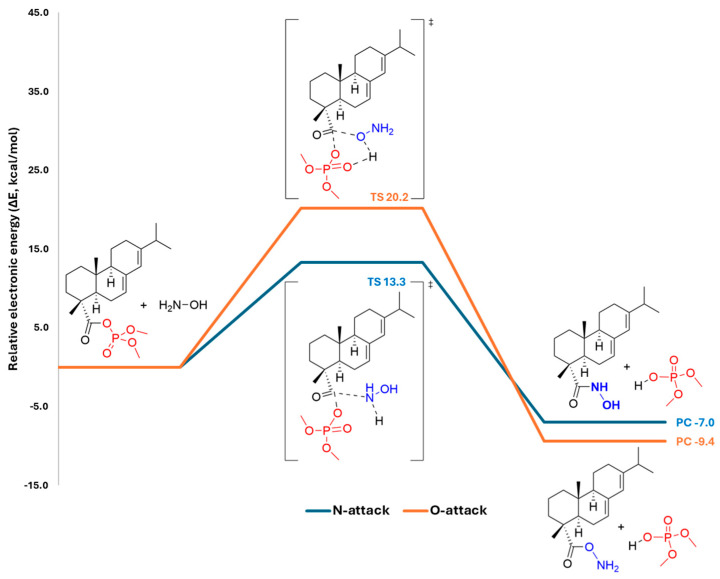
Computed free-energy profile (M06-2X/6-31G**/PCM (DMF)) for the competitive N-attack versus O-attack of hydroxylamine on the activated phosphate of abietic acid (**1**).

Finally, the standardized heating to 40 °C was validated as optimized for the one-pot protocol, ensuring the full solubility of the hydroxylamine hydrochloride in DMF. As demonstrated by our computational analysis, this energy input allows the system to easily overcome the calculated activation barrier of 13.3 kcal/mol (Figure 2), facilitating access to the transition state without compromising the stability of the activated intermediates.

### 2.3. Mechanistic Rationalization via DFT Studies

To gain deeper mechanistic insight into the observed chemoselectivity of the hydroxyamidation reaction, we investigated the competitive nucleophilic acyl substitution pathways occurring in the second stage of the one-pot process. While the global reaction starts from abietic acid (**1**), the computational study specifically evaluated the energy profiles for both routes, N-attack and O-attack, starting from the activated phosphate mixed anhydride as the common reactive precursor (Figure 2). This intermediate is formed in situ during the first activation step and serves as the branching point for the subsequent nucleophilic attack by hydroxylamine.

For the hydroxamic amide pathway, the calculated energy variation (∆E) for the RC (reactant complex), TS (transition state), and PC (product complex) presents a profile of 0.0, +10.8, and −10.0 kcal/mol relative to its initial association complex, as detailed in Figure S17. This activation barrier of 10.8 kcal/mol is relatively low. While the inclusion of entropic factors (∆S) will expectedly increase this value, the overall exothermic nature of the process suggests that amide formation is unlikely to be reversible under the employed experimental conditions. Conversely, the route leading to the amino ester via oxygen attack exhibits a profile of 0.0, +20.2, and −9.4 kcal/mol relative to its own initial complex, as illustrated in Figure S18.

However, to provide a comprehensive and comparable view of the chemoselectivity during this second step, a common energy reference point must be established, considering that both initial reactant complexes exist in a rapid exchange equilibrium. A comparison of the total electronic energies (HF) reveals that the ester-type reactant complex is lower in energy (HF = –1708.1497 a.u., see Table S6) than its amide-type counterpart (HF = –1708.1449 a.u., see Table S3). Since the ester-type complex is the more stable of the two, it was selected as the global zero-energy reference for Figure 2. Under this unified framework, the profile for amide formation is adjusted to 0.0, +13.3, and −7.0 kcal/mol, while the ester pathway remains at 0.0, +20.2, and −9.4 kcal/mol.

These results reveal a clear dichotomy between kinetic and thermodynamic control in the functionalization of the abietane skeleton. From a thermodynamic perspective, the amino ester product is more stable than the hydroxy amide by 2.4 kcal/mol. Nevertheless, the reaction is strictly governed by kinetic control, as the activation barrier for N-attack is 6.9 kcal/mol lower than that required for O-attack. At room temperature (298.15 K), this energetic difference favors amide formation by approximately five orders of magnitude in the rate constant ratio. Such kinetic preference provides a compelling rationalization for the exclusive formation of the hydroxamic acid observed experimentally.

The second stage of the reaction was conducted at 40 °C to ensure the complete solubility of the hydroxylamine hydrochloride in DMF. This temperature ensures a high reaction rate while remaining sufficiently low to prevent the thermal decomposition of the sensitive activated phosphate intermediate.

Finally, Intrinsic Reaction Coordinate (IRC) calculations confirmed that the transition state for the amide pathway connects the activated phosphate reactant specifically to the desired hydroxamic product. These findings validate a mechanistic framework where the steric hindrance of the abietane skeleton is overcome by the kinetic accessibility of the N-attack route.

## 3. Materials and Methods

### 3.1. Materials and Equipment

All reagents were obtained from commercial sources and used without further purification unless otherwise noted. Abietic acid (95% purity) was purchased from Biosynth. (Staad, Switzerland). Despite dehydroabietic acid (DHA) being commercially available, it was synthesized under similar conditions to the procedure reported by Halbrook and Lawrence [17], from an old aged sample of abietic acid (ca. 50–60% purity) we had available in the laboratory, by disproportionation. To this end, 1% (*w*/*w*) of 10% Pd on carbon (300 mg) (Merck Life Science S.L.U., St. Louis, MO, USA) were added to 30 g of impure abietic acid and heated at 240 °C for 4 h without a stream of nitrogen gas. It was cooled to around 50 °C and diluted with 50 mL of ethyl acetate and filtered through a paper filter. After removal of the solvent the crude product (26 g) was chromatographed on silica eluting with hexane-ethyl acetate 7:3 to give 9.6 g of DHA which contained ca. 10% of an impurity 6,7-dehydrodehydroabietic acid. Further purification was attempted by making 2-aminoethanol salt and crystallizing [17]. For this purpose, 9.6 g of impure DHA were dissolved in 5 mL of 96% ethanol at 80 °C with continuous stirring and 1.8 mL of ethanolamine (Merck Life Science S.L.U., St. Louis, MO, USA) and 5 mL of pre-heated water were added. Without cooling, it was extracted once with 7 mL of isooctane (Merck Life Science S.L.U., St. Louis, MO, USA) but we were unable to do more hot extractions with isooctane like Halbrook and Lawerence since the product rapidly precipitated. The salt was collected and washed with cold 1:1 (*v*/*v*) ethanol-water (50 mL) and air dried overnight. The resulting salt (9.8 g) was recrystallized once, dissolved with 20 mL of 96% ethanol at 70 °C and 21 mL of water used as anti-solvent, allowed to cool down to rt with a crystal seed. An amount of 5 g of salt was obtained as a white solid with trace brownish powder. This salt was dissolved in 10 mL of 96% ethanol at 70 °C and acidified to pH = 1 by dropwise addition of 1 mL of conc. aqueous HCl. The resulting white precipitate, after cooling to rt, was filtered off and washed with water and air dried at rt overnight to afford 3.7 g of DHA which still contained the ca. 10% impurity of the double bond between C6-C7. Finally, 3.2 g of this impure DHA was hydrogenated using 10% Pd/C (210 mg) in MeOH (60 mL) under 1 atm of hydrogen for 20 h. After filtration through a pad of celite under reduced pressure and washing with 40 mL of MeOH, the clear filtrate was concentrated to give 3.04 g of pure DHA as a white solid.

Diethyl chlorophosphate (DCP, 97%), triethylamine (Et_3_N, 99%), and hydroxylamine hydrochloride (NH_2_OH·HCl, 99%) were obtained from Merck Life Science (St. Louis, MO, USA). Anhydrous N,N-dimethylformamide (DMF) was purchased from ACROS Organics (Geel, Belgium) (extra dry over molecular sieves, AcroSeal). All moisture-sensitive reactions were performed under an argon atmosphere using an argon-filled balloon.

Analytical TLC was performed on silica gel 60 F_254_ plates, and column chromatography was carried out on silica gel 60 (0.040–0.063 mm) (Merck Life Science S.L.U., St. Louis, MO, USA). Hydroxamic acids can be visualized on TLC plates as reddish spots by using 5% aqueous FeCl_3_ solution as staining reagent. Melting points were determined on a Cole-Parmer MP-800D apparatus (Vernon Hills, IL, USA). Optical rotations were measured on a JASCO P2000 polarimeter (Tokyo, Japan). ^1^H and ^13^C NMR spectra were recorded on a Bruker Ascend 400 spectrometer (Billerica, MA, USA) at 400 and 100 MHz, respectively, using CDCl_3_ as solvent. Chemical shifts (δ) are reported in ppm relative to internal CDCl_3_ (δ_H_ 7.26 and δ_C_ 77.00). High-resolution mass spectra (HRMS) were obtained on an AB Sciex QTOF 6600+ mass spectrometer (Framingham, MA, USA). Elemental analyses (C, H, N) were performed with a Thermo Scientific™ Flash Smart™ analyzer (Waltham, MA, USA), using sulfanilamide as reference. The UV-Vis measurements were performed in solid in a Cary 7000 spectrophotometer (Agilent, Santa Clara, CA, USA) in the range between 190 and 800 nm.

### 3.2. General Procedure for the Synthesis of N-Hydroxy-Abieta-7,13-dien-18-amide (***1a***, Abietohydroxamic Acid)

To a cooled solution of abietic acid (93% purity estimated by ^1^H, 649 mg, 2.0 mmol), Et_3_N (2.54 mL, 18.0 mmol) in anhydrous DMF (12 mL) at 0 °C under Ar atmosphere, diethyl chlorophosphate (DCP) (595 µL, 4.0 mmol) was added dropwise and allowed to warm to rt. After being stirred for 7 h, it was added NH_2_OH·HCl (702 mg, 10.0 mmol) in one portion. The resulting reaction mixture was heated at 40 °C overnight (16 h). Then, it was diluted with H_2_O (5 mL) and extracted with ethyl acetate (4 × 20 mL). The combined organic extracts were washed with 1N HCl (2 × 5 mL), H_2_O (5 mL), brine (5 mL), dried (Na_2_SO_4_) and concentrated. The crude residue (2.52 g, yellow solution, estimation by ^1^H NMR ca. 3.3:1 hydroxamic acid:carboxylic acid) was chromatographed on silica eluting with n-hexane-ethyl acetate (6:4) to give 101.7 mg of recovered abietic acid followed by 410 mg (64.6% yield, estimated 77.7% yield based on recovered starting material (brsm)) of abietohydroxamic acid **1a** as a white solid: m.p. 125–128 °C (lit. [11], 127–131 °C); ${\left[\alpha \right]}_{25}^{D}$ = −72.9° (c 0.5, DCM) (lit. [11], ${\left[\alpha \right]}_{20}^{D}$ = −78 °); UV-Vis λ_max_ 226 nm; ^1^H NMR (CDCl_3_, 400 MHz) δ_H_: 5.75 (1H, s), 5.32 (1H, br s), 2.21 (1H, sept., *J* = 6.8), 2.08–1.90 (4H, m), 1.88–1.77 (3H, m), 1.60–1.50 (4H, m), 1.23 (3H, s), 1.25–1.20 (3H, m), 1.00 (3H, d, *J* = 6.8), 0.99 (3H, d, *J* = 6.8), 0.82 (3H, s); ^13^C NMR (CDCl_3_, 100 MHz) δ_C_: 176.6 (s), 145.3 (s), 135.5 (s), 122.3 (d), 120.1 (d), 50.9 (d), 45.2 (d), 45.1 (s), 38.1 (t), 37.2 (t), 34.9 (d), 34.6 (s), 27.4 (t), 25.2 (t), 22.4 (t), 21.4 (q), 20.8 (q), 17.9 (t), 15.8 (q), 14.2 (q); HRMS (ESI) *m*/*z* 318.2425 [M + H]^+^, calcd for C_20_H_32_NO_2_: 318.2433; Anal. calcd. for C_20_H_31_NO_2_: C, 75.7; H, 9.8; N, 4.4 Found: C, 75.2; H, 10.1; N, 4.4.

### 3.3. Synthesis of N-Hydroxy-Abieta-8,11,13-trien-18-amide (***2a***, Dehydroabietohydroxamic Acid)

Following the general procedure described in Section 3.2, dehydroabietic acid (600 mg, 2.0 mmol) was treated with DCP and then with NH_2_OH·HCl. After stirring overnight (16 h) at 40 °C, the reaction was processed using the optimized low-volume work-up (5 mL H_2_O) to yield the crude residue (2.55 g, orangish solution, estimated ^1^H NMR ratio ca. 3.3:1 hydroxamic acid:carboxylic acid). Purification by column chromatography eluting with n-hexane-ethyl acetate (6:4) yielded 465.1 mg (73.8% yield, 93.5% brsm) of dehydroabietohydroxamic acid **2a** as a white solid: mp 123–125 °C; ${\left[\alpha \right]}_{21}^{D}$ = +38.2° (c 1.0, DCM); UV-Vis λ_max_ 228, 269, 277 nm; ^1^H NMR (CDCl_3_, 400 MHz) δ_H_ 7.15 (1H, d, *J* = 8.4), 6.99 (1H, dd, *J* = 8.4, 2.4), 6.86 (1H, br s), 2.89–2.85 (2H, m), 2.82 (1H, sept., *J* = 6.8), 2.30 (1H, br d, *J* = 12.6), 2.21 (1H, dd, *J* = 12.6, 2.2), 1.89–1.69 (4H, m), 1.58–1.53 (1H, m), 1.51–1.45 (2H, m), 1.25 (3H, s), 1.22 (6H, d, *J* = 6.8), 1.22 (3H, s); ^13^C NMR (CDCl3, 100 MHz) δ_C_ 176.7 (s), 146.6 (s), 145.8 (s), 134.5 (s), 126.8 (d), 124.0 (d), 123.9 (d), 46.2 (s), 44.9 (d), 37.7 (t), 37.0 (s), 36.8 (t), 33.4 (d), 29.8 (t), 25.2 (q), 24.0 (q), 24.0 (q), 21.0 (t), 18.3 (t), 15.4 (q); HRMS (ESI) *m*/*z* 316.2264 [M + H]^+^, calcd for C_20_H_30_NO_2_: 316.2277; Anal. calcd. for C_20_H_29_NO_2_: C, 76.2; H, 9.3; N, 4.4 Found: C, 75.7; H, 9.0; N, 4.2.

### 3.4. Isolation and Characterization of the Diethyl Phosphate Mixed Anhydride Intermediate of Dehydroabietic Acid (***Int2***)

Following the experimental conditions described in Table 1 (Entry 4), the intermediate was isolated for mechanistic validation.

Diethyl (abieta-8,11,13-trien-18-oyl) phosphate (**Int2**, Scheme 2b). Colorless oil; ^1^H NMR (CDCl_3_, 400 MHz) δ_H_: 7.16 (1H, d, *J* = 8.0), 7.01 (1H, d, *J* = 8.4, 2.0), 6.89 (1H, br s), 4.30–4.20 (4H, m), 2.95–2.87 (2H, m), 2.82 (1H, sept., *J* = 6.8), 2.34–2.29 (1H, m), 2.19 (1H, dd, *J* = 12.4, 2.0), 1.92–1.71 (5H, m), 1.58–1.46 (2H, m), 1.35 (6H, tt, *J* = 7.2, 0.8), 1.31 (3H, s), 1.22 (6H, d, *J* = 7.2), 1.21 (3H, s); ^13^C NMR (CDCl_3_, 100 MHz) δ_C_: 172.9 (d, *J*_C-P_ = 10.8), 146.3 (s), 145.9 (s), 134.5 (s), 126.6 (d), 124.1 (d), 124.0 (d), 65.0 (d, *J*_C-P_ = 5.7), 64.9 (d, *J*_C-P_ = 5.7), 48.8 (d, *J*_C-P_ = 5.7), 44.7 (d), 37.7 (t), 36.9 (s), 35.8 (t), 33.4 (q), 29.9 (t), 25.1 (d), 23.9 (q), 23.9 (q), 21.6 (t), 18.3 (t), 16.4 (q), 16.1 (q), 16.0 (q).

### 3.5. Computational Methods

All quantum-chemical calculations were performed using the Gaussian 09 software package (Revision D.01). Geometry optimizations and frequency calculations were carried out at the M06-2X/6-31G** level of theory, which is widely validated for describing thermochemistry and activation barriers in organic reactions [18]. Solvent effects were incorporated implicitly through the Polarizable Continuum Model (PCM) using N,N-dimethylformamide (DMF, ε = 37.22) as the solvent, consistent with the experimental reaction conditions.

To reduce computational costs while preserving the stereoelectronic features of the sterically congested abietane skeleton, a simplified model system was employed. In this model, dimethoxyphosphate groups replaced the full diethyl phosphate substituents, yielding a system that preserves the reactive environment at the C-18 center.

Stationary points on the potential energy surface were located using analytical gradient optimization. Transition state (TS) structures were obtained using the Berny algorithm and were initially identified through relaxed potential energy surface (PES) scans along the forming N–C(=O) or O–C(=O) bond coordinates. The nature of all stationary points was confirmed by harmonic frequency analysis computed analytically at the same level of theory. Local minima displayed zero imaginary frequencies, while each transition state exhibited exactly one imaginary frequency corresponding to the normal mode of the bond-forming event.

Intrinsic Reaction Coordinate (IRC) calculations were performed for each TS to rigorously confirm the connectivity between the transition states and the corresponding reactants and products. Electronic energy variations (∆E) are reported for the individual pathways relative to their respective reactant complexes to describe the intrinsic barriers. For the global mechanistic discussion and the rationalization of chemoselectivity, all relative energies are referenced to the most stable ester-type reactant complex (R) set to 0.0 kcal/mol.

## 4. Conclusions

We have developed a practical single-vessel protocol for the synthesis of abietane-type hydroxamic acids. This methodology successfully overcomes the important steric hindrance associated with the C18 carboxyl group. The use of diethyl chlorophosphate (DCP) as an activating agent proved key in comparison to traditional strategies, as it facilitates the formation of reactive diethyl phosphate mixed anhydride intermediates. These species were successfully isolated and characterized, providing direct experimental evidence of the activation pathway.

A fundamental contribution of this study is the optimization of the isolation process to handle the amphiphilic nature of the target products. By reducing the aqueous volume to 5 mL (for 2 mmol of starting acid) during the work-up, we effectively prevented product loss into the aqueous phase. This modification allowed isolated yields to increase from initial values of 17.3–34.2% to 64.6% from abietic acid and 73.8% from dehydroabietic acid. We concluded that the deliberate retention of a small amount of residual DMF in the crude material was a necessary strategic decision to ensure maximum product recovery. Crucially, this residual solvent did not interfere with the subsequent flash chromatography purification.

Furthermore, DFT calculations provided a comprehensive rationalization for the exclusive formation of N-hydroxy amides. The process is governed by strict kinetic control. Although the amino ester is the thermodynamically more stable product, the formation of the hydroxamic amide is favored by an activation barrier that is 6.9 kcal/mol lower than the oxygen-attack route. The application of 40 °C ensured the solubility of reagents and the stability of the activated intermediates. This integrated approach establishes a reliable and scalable methodology for the chemical valorization of abundant natural terpenoids into potential bioactive scaffolds.

## Data Availability

The data is available in the manuscript and further details or samples of **1a** or **2a** upon request.

## Supplementary Materials

The following supporting information can be downloaded at: https://www.mdpi.com/article/10.3390/molecules31101637/s1, Figures S1–S11: copies of 1H, 13C, DEPT NMR spectra of **1a**, **2a** and **int2**; Figures S12–S15: copies of HRMS and UV spectra of **1a** and **2a**; Figures S16–S18: Optimized structures and relative energies for the species; Tables S1–S8: Cartesian coordinates; Video animation of reaction path.
