# Supplementary material for: One-Pot Synthesis of Abietane-Type Hydroxamic Acids: Process Optimization and Mechanistic Insights
Source: Molecules. 2026 May 13;31(10):1637. doi: 10.3390/molecules31101637 (PMC13210091; doi:10.3390/molecules31101637)
Supplement: Supplementary file 1 [file molecules-31-01637-s001.zip › molecules-4302922-supplementary.pdf]

Supplementary Information

One-Pot Synthesis of Abietane-Type Hydroxamic Acids: Pro-cess Optimization and Mechanistic Insights

William E. Mendoza-Hernández [1], Ramón J. Zaragozá [2], Urbano Díaz [1], and Miguel A. González-Cardenete [1,*]

[1] Instituto de Tecnología Química, Consejo Superior de Investigaciones Cientificas- Universitat Politècnica de València, 46022 Valencia, Spain

[2]Departamento de Química Orgánica, Universitat de Valencia, Dr. Moliner 50, 46100 Burjassot, Valencia, Spain

*Correspondence: migoncar@itq.upv.es (M.A.G.-C.)

Content:

Part A: Characterization Spectra

Figure S1. ^{1}H NMR spectrum of abietohydroxamic acid (**1a**).

Figure S2. ^{13}C NMR spectrum of abietohydroxamic acid (**1a**).

Figure S3. DEPT135 spectrum of abietohydroxamic acid (**1a**).

Figure S4. DEPT90 spectrum of abietohydroxamic acid (**1a**).

Figure S5. ^{1}H NMR spectrum of dehydroabietohydroxamic acid (**2a**).

Figure S6. ^{13}C NMR spectrum of dehydroabietohydroxamic acid (**2a**).

Figure S7. DEPT135 spectrum of dehydroabietohydroxamic acid (**2a**).

Figure S8. DEPT90 spectrum of dehydroabietohydroxamic acid (**2a**).

Figure S9. ^{1}H NMR spectrum of diethyl phosphate mixed anhydride of dehydroabietic acid (**Int2**)

Figure S10. ^{13}C NMR spectrum of diethyl phosphate mixed anhydride of dehydroabietic acid (**Int2**)

Figure S11. DEPT135 spectrum of diethyl phosphate mixed anhydride of dehydroabietic acid (**Int2**)

Figure S12. High-Resolution Mass Spectra (HRMS) of abietohydroxamic acid (**1a**): (a) Full mass spectrum showing the parent ion cluster; (b) Comparison between the experimental isotopic pattern and the calculated theoretical distribution.

Figure S13. High-Resolution Mass Spectra (HRMS) of dehydroabietohydroxamic acid (**2a**): (a) Full mass spectrum showing the parent ion cluster; (b) Comparison between the experimental isotopic pattern and the calculated theoretical distribution.

Figure S14. UV-Vis spectrum (Kubelka-Munk) of abietohydroxamic acid (**1a**).

Figure S15. UV-Vis spectrum (Kubelka-Munk) of dehydroabietohydroxamic acid (**2a**).

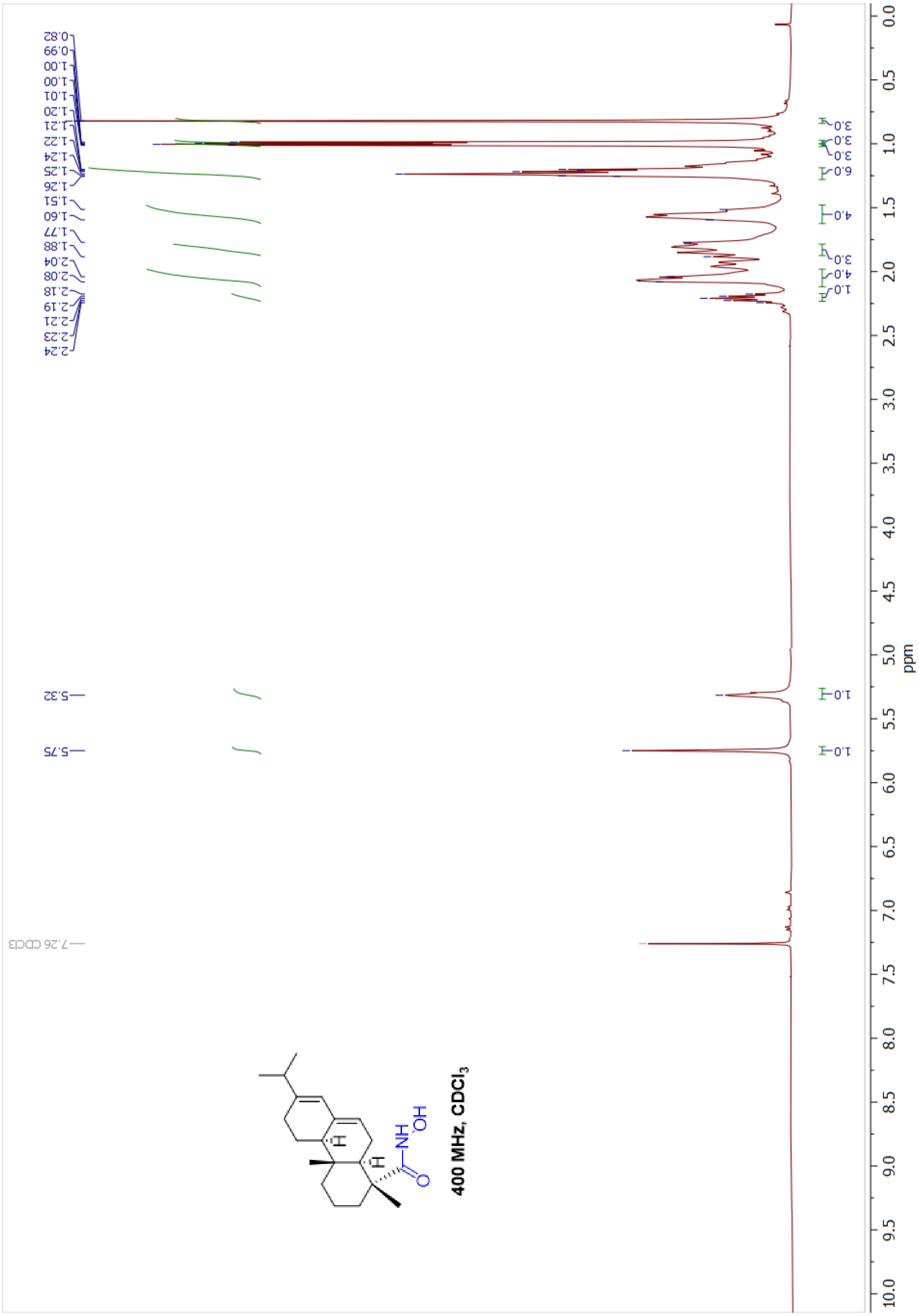

Figure S1. ^{1}H NMR spectrum of abietohydroxamic acid (**1a**).

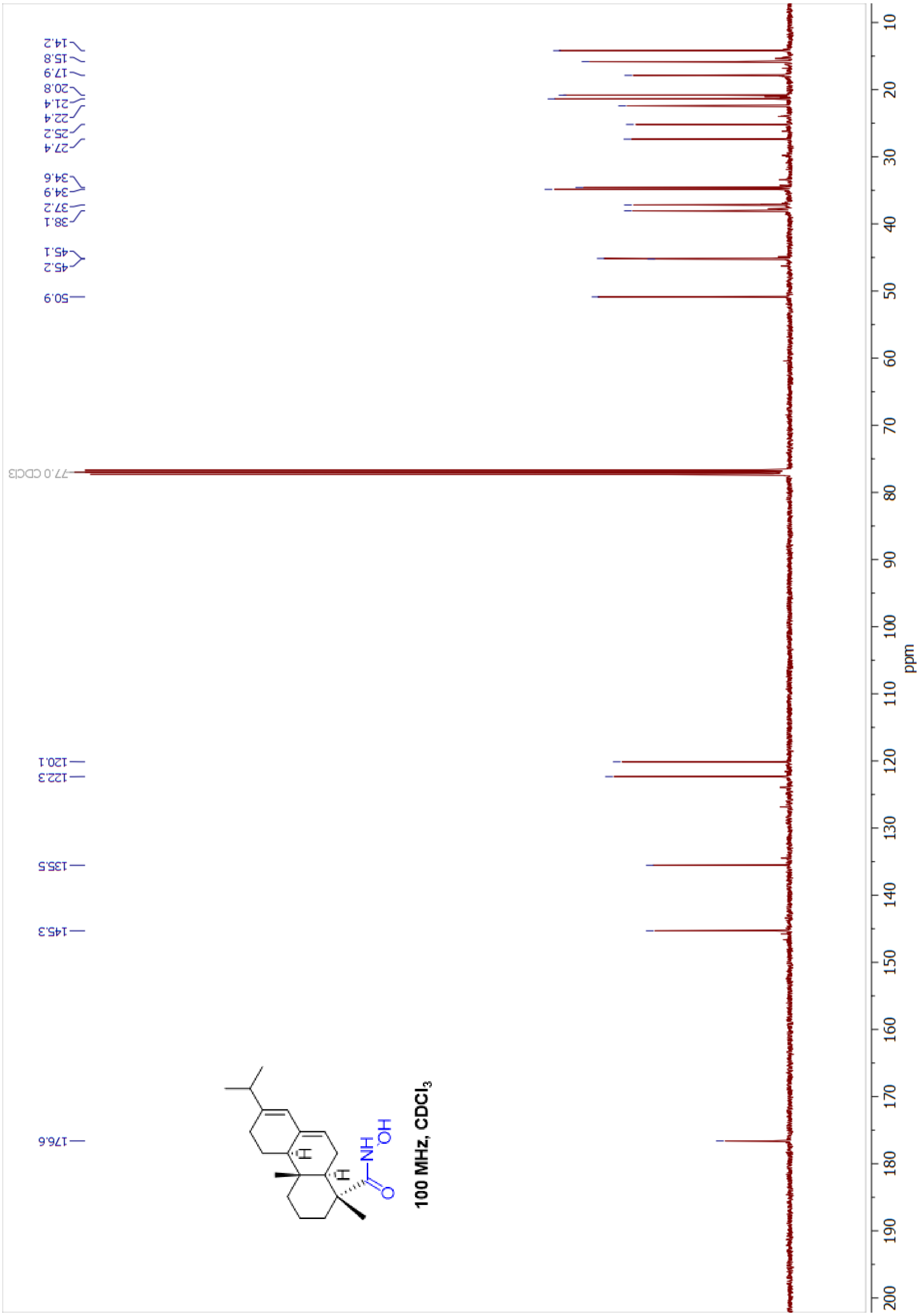

Figure S2. ^{13}C NMR spectrum of abietohydroxamic acid (**1a**).

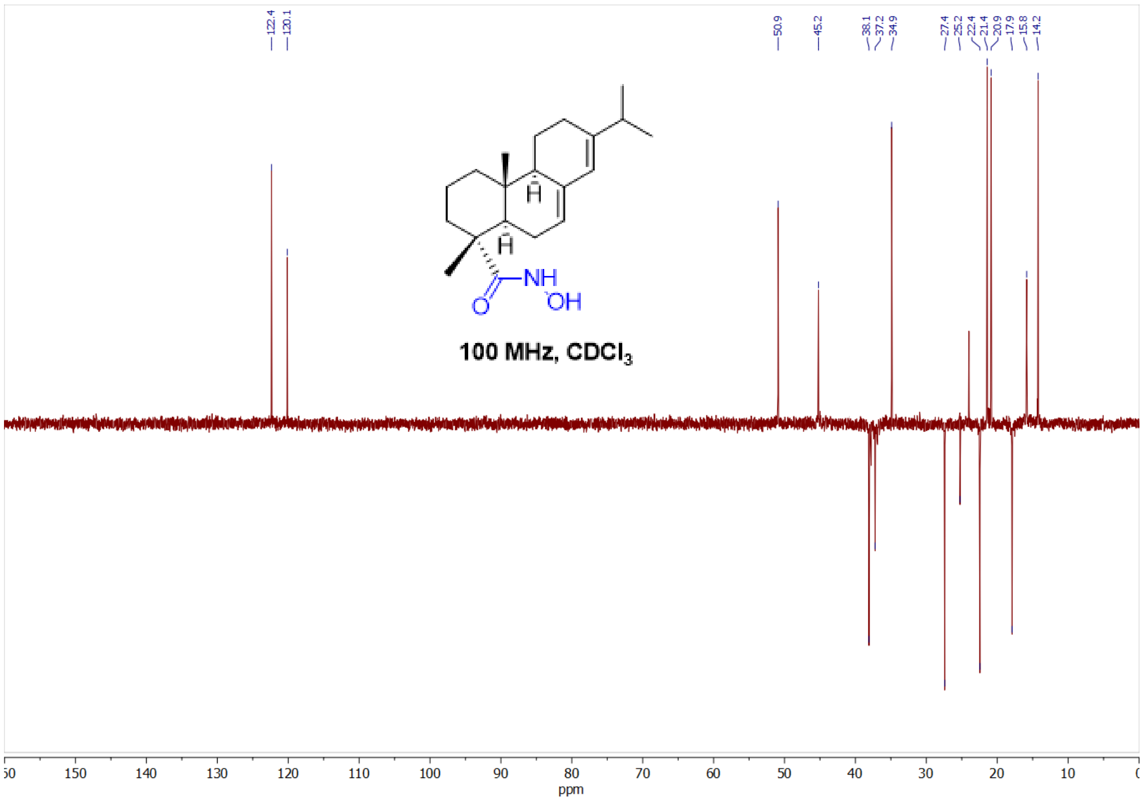

Figure S3. DEPT135 spectrum of abietohydroxamic acid (**1a**).

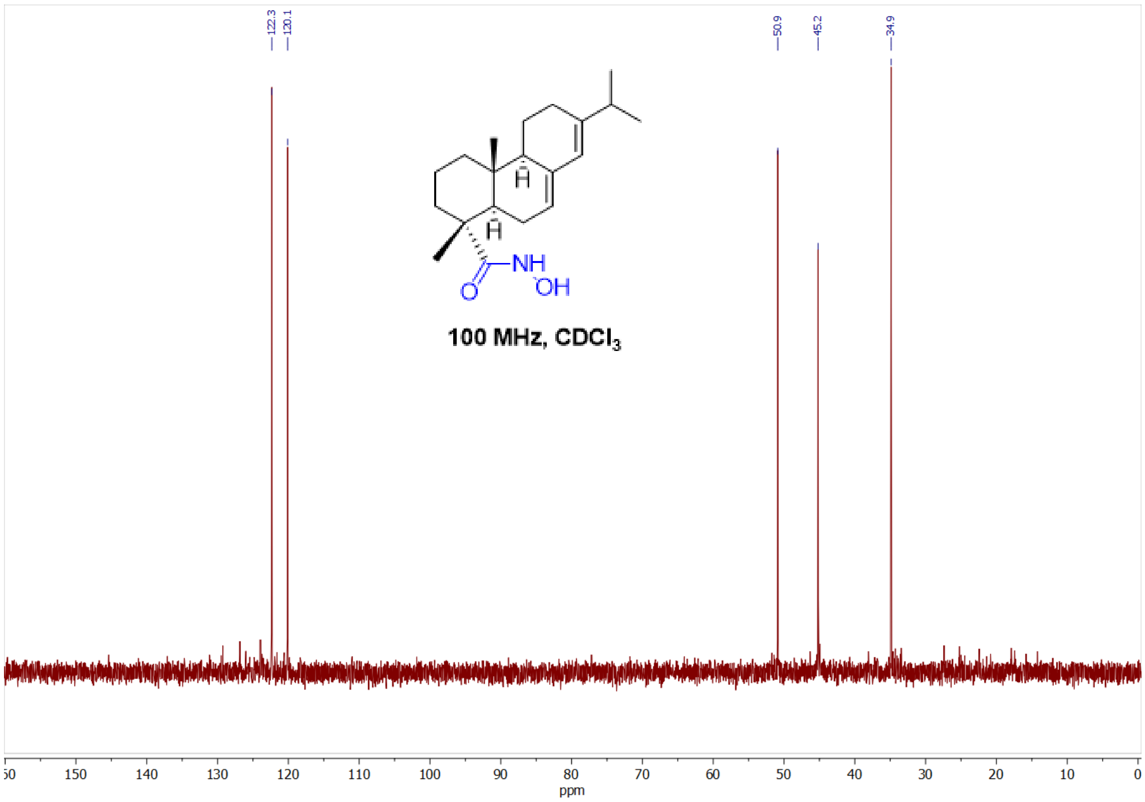

Figure S4. DEPT90 spectrum of abietohydroxamic acid (**1a**).

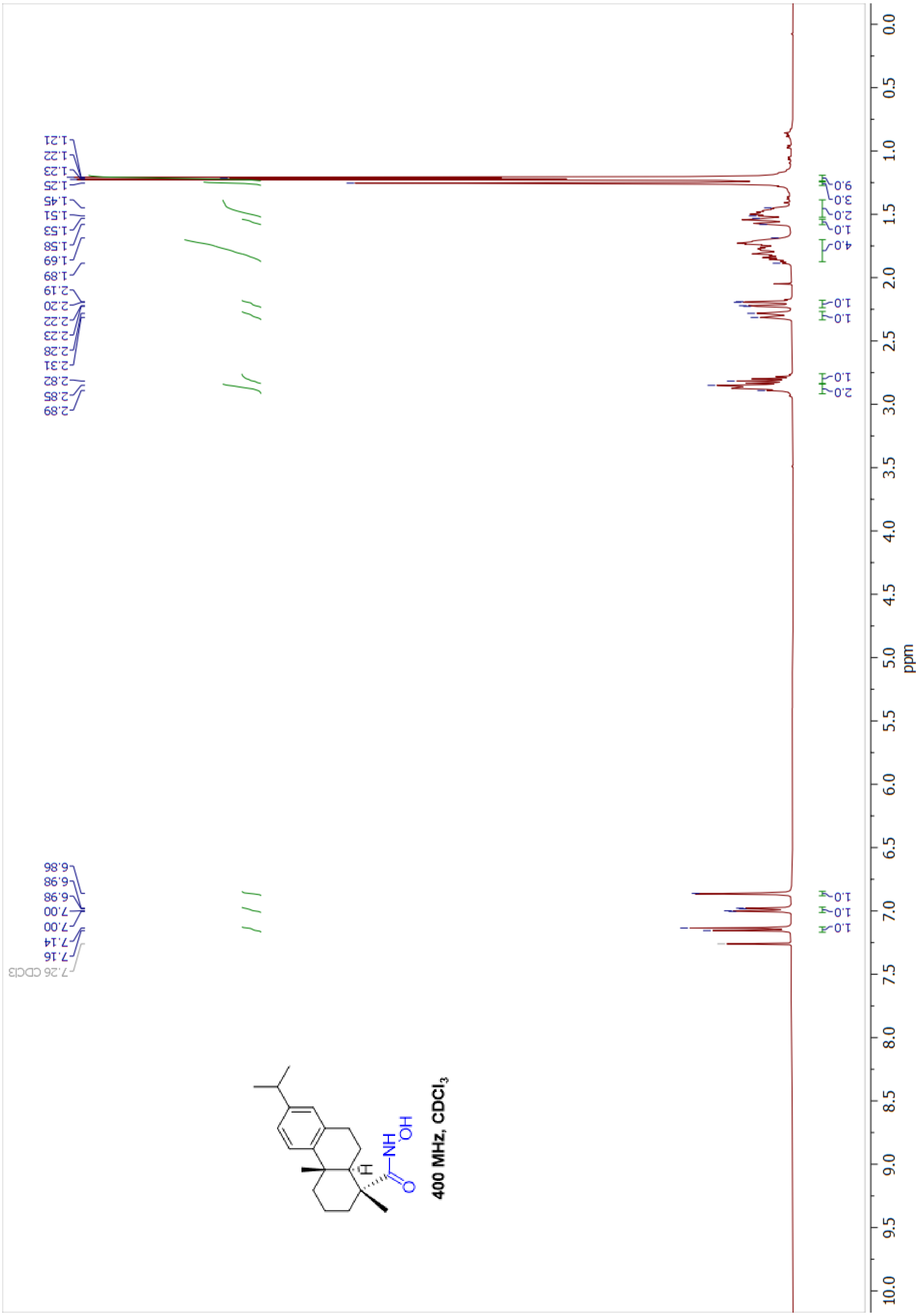

Figure S5. ^{1}H NMR spectrum of dehydroabietohydroxamic acid (**2a**).

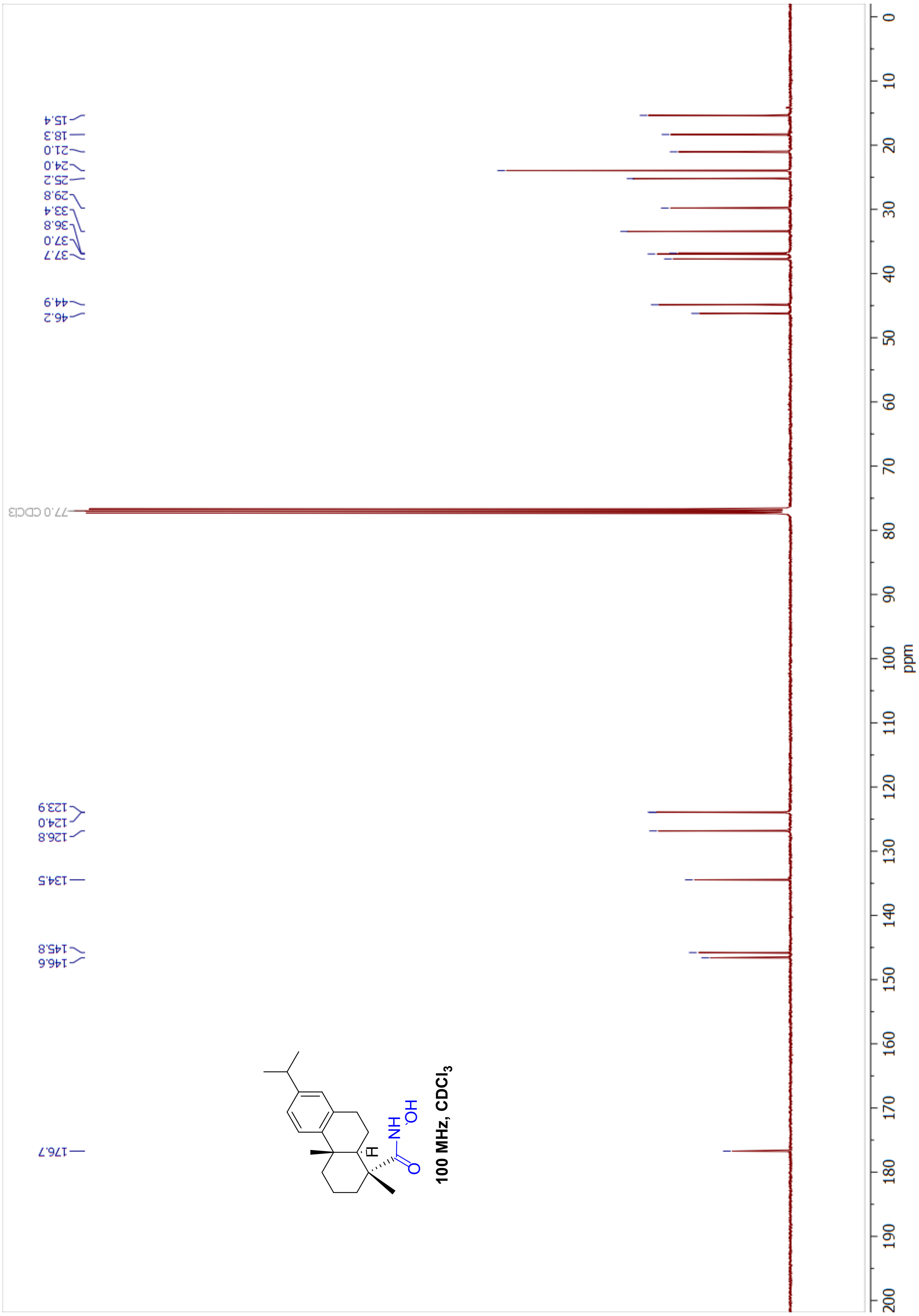

Figure S6. ^{13}C NMR spectrum of dehydroabietohydroxamic acid (**2a**).

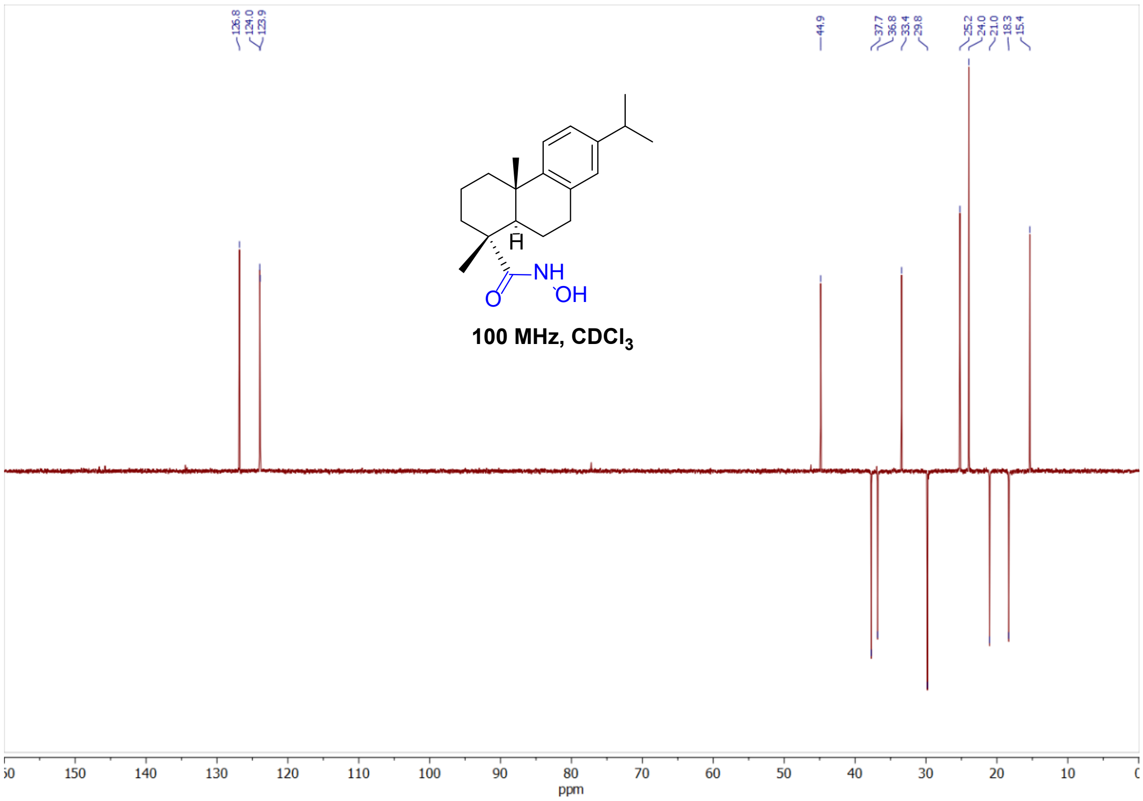

Figure S7. DEPT135 spectrum of dehydroabietohydroxamic acid (**2a**).

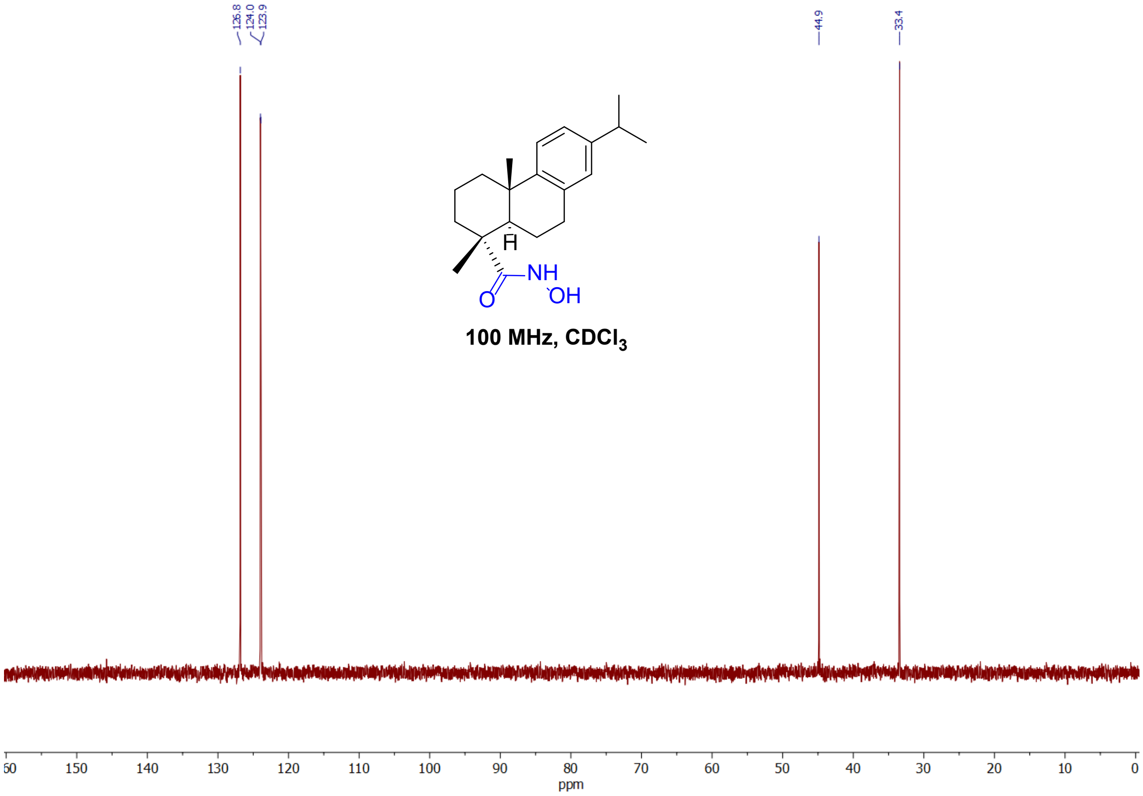

Figure S8. DEPT90 spectrum of dehydroabietohydroxamic acid (**2a**).

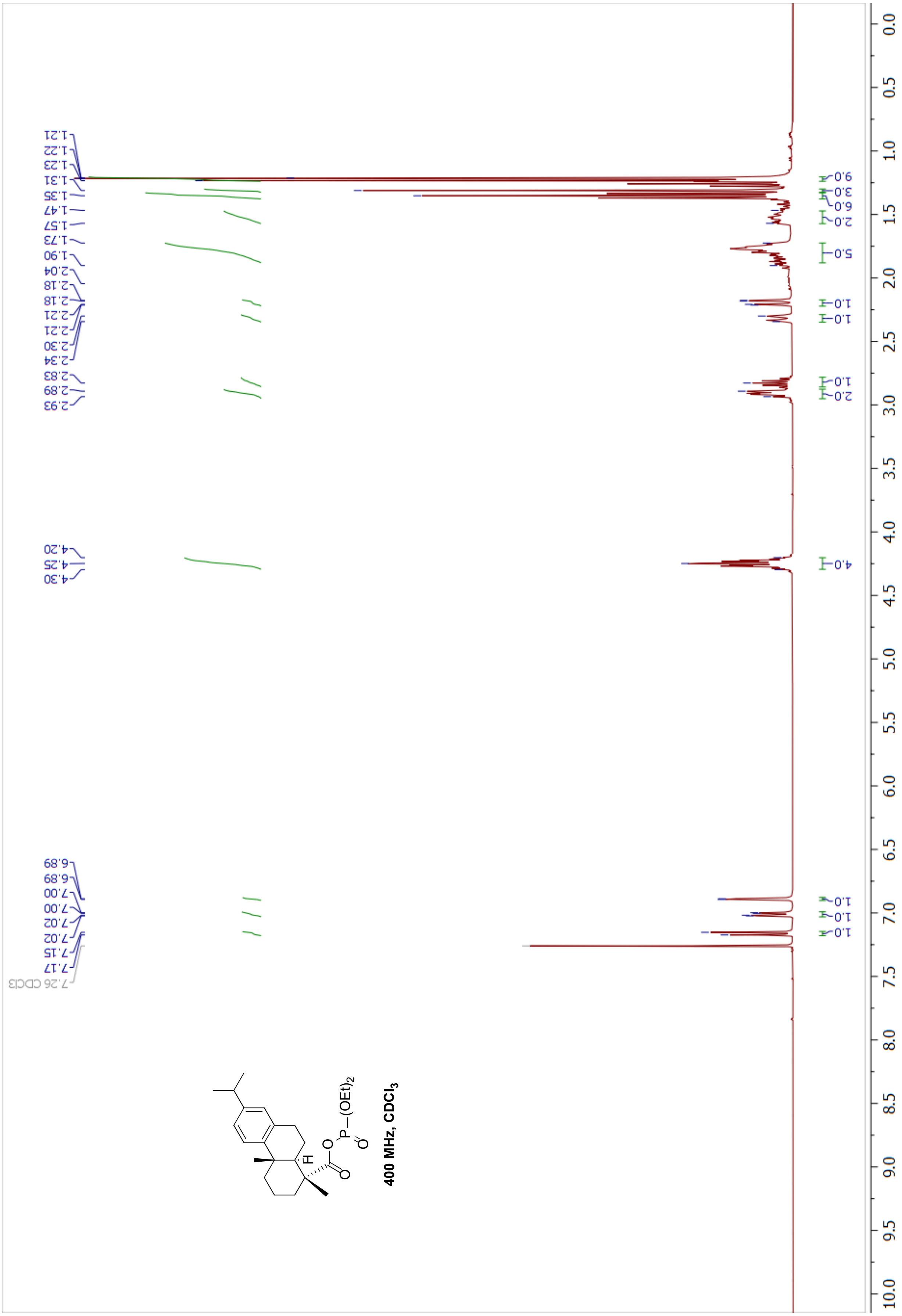

Figure S9. ^{1}H NMR spectrum of diethyl phosphate mixed anhydride of dehydroabietic acid (**Int2**)

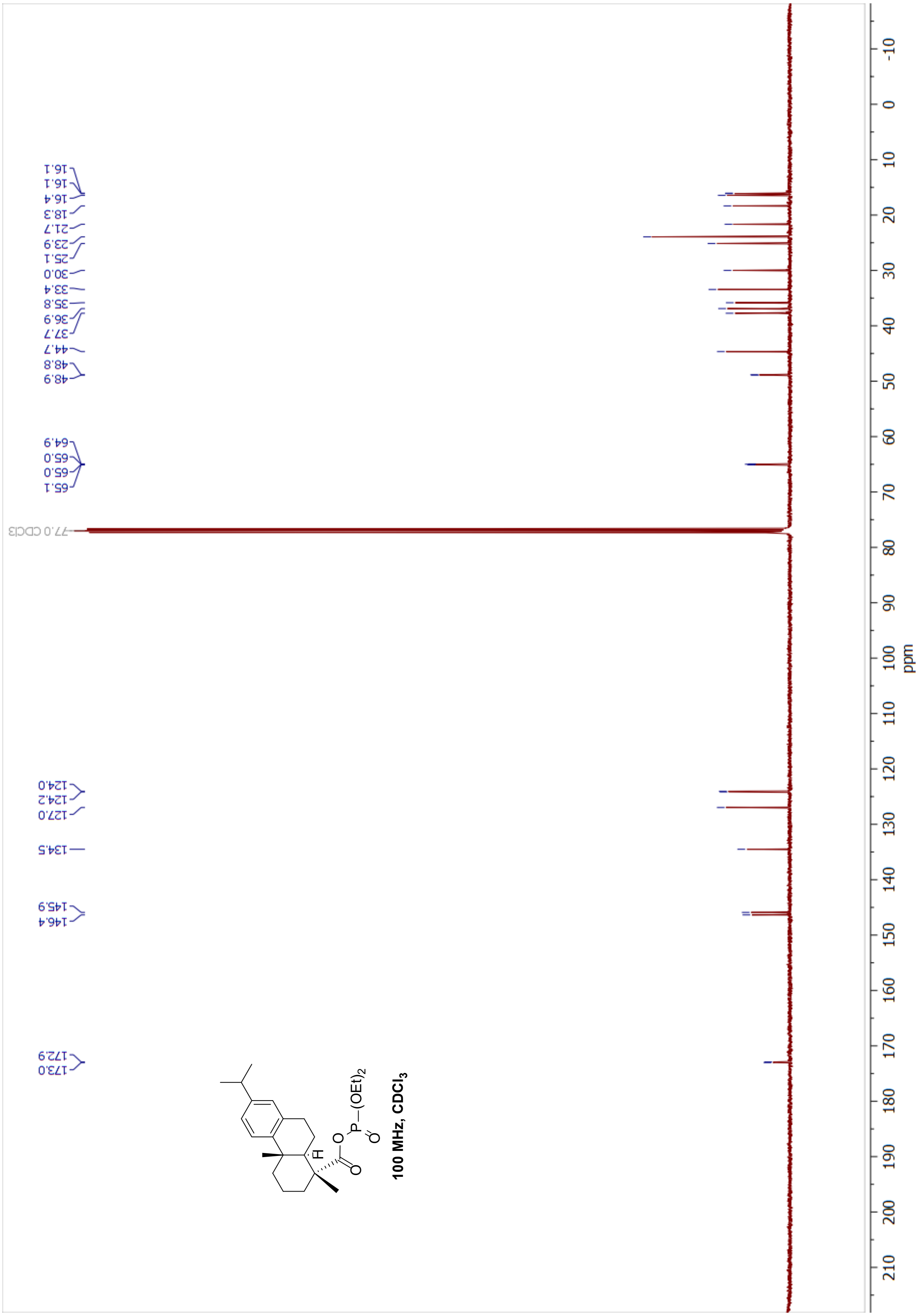

Figure S10. ^{13}C NMR spectrum of diethyl phosphate mixed anhydride of dehydroabietic acid (**Int2**)

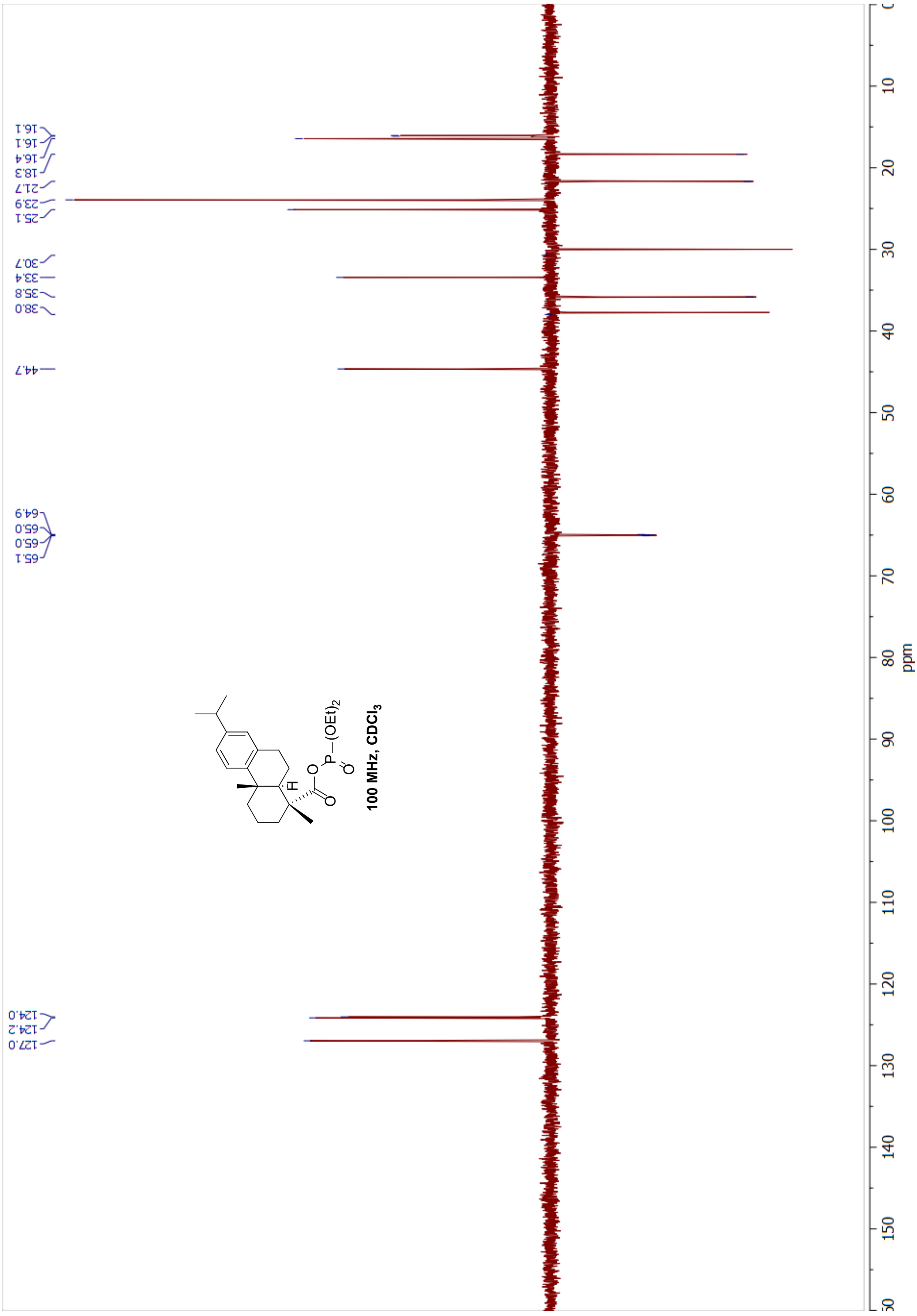

Figure S11. DEPT135 spectrum of diethyl phosphate mixed anhydride of dehydroabietic acid (**Int2**)

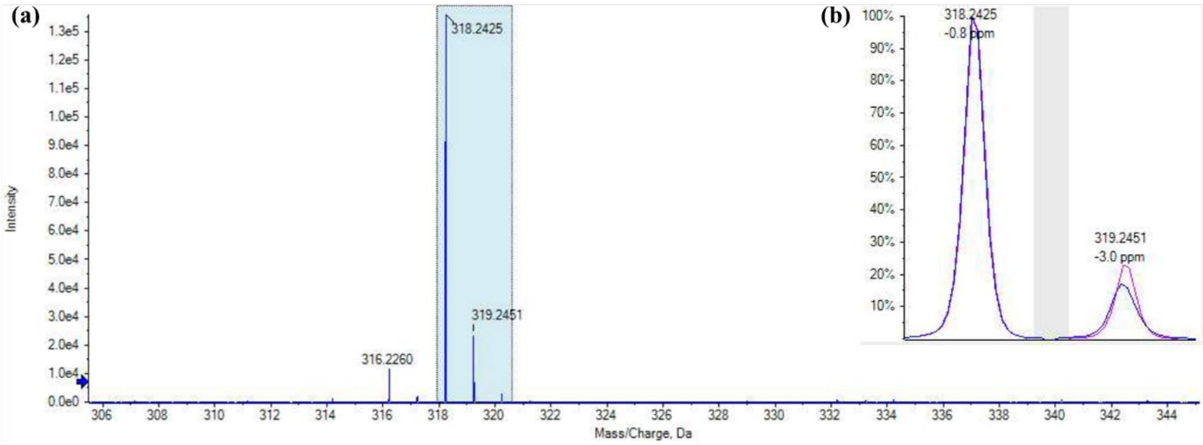

Figure S12. High-Resolution Mass Spectra (HRMS) of abietohydroxamic acid (**1a**): (a) Full mass spectrum showing the parent ion cluster; (b) Comparison between the experimental isotopic pattern and the calculated theoretical distribution.

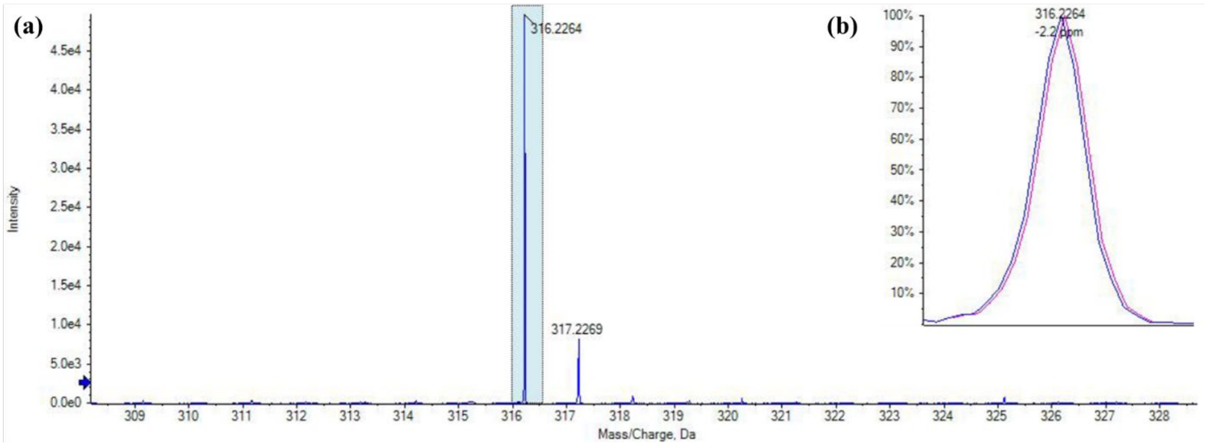

Figure S13. High-Resolution Mass Spectra (HRMS) of dehydroabietohydroxamic acid (**2a**): (a) Full mass spectrum showing the parent ion cluster; (b) Comparison between the experimental isotopic pattern and the calculated theoretical distribution.

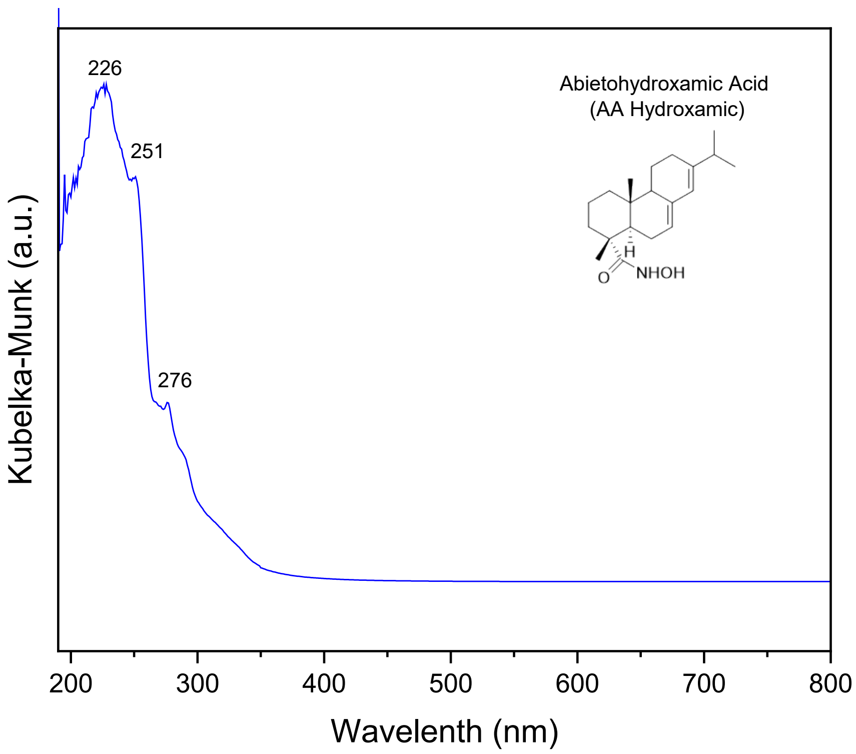

Figure S14. UV-Vis spectrum (Kubelka-Munk) of abietohydroxamic acid (**1a**).

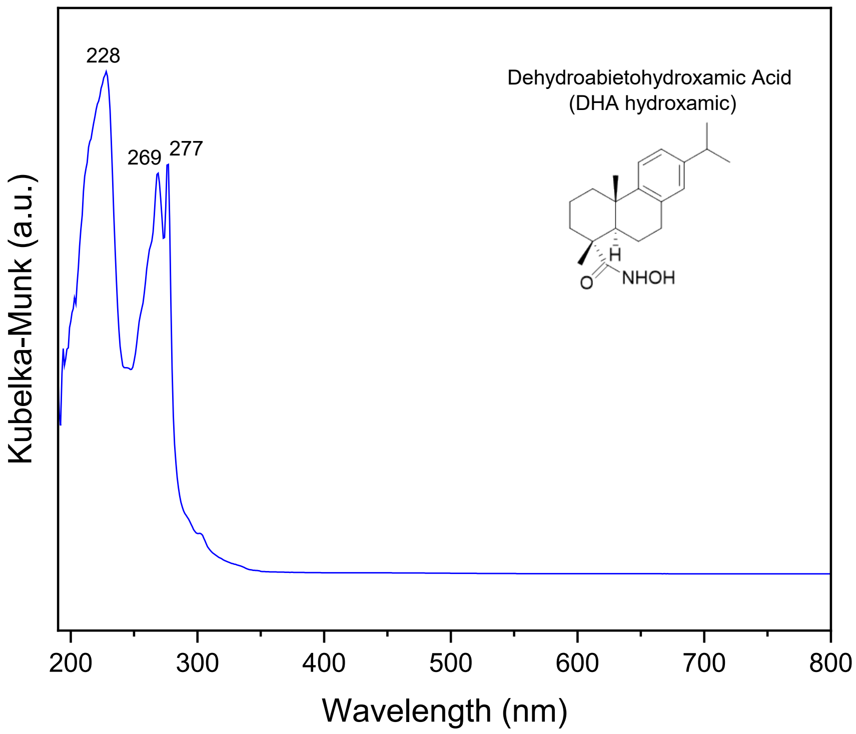

Figure S15. UV-Vis spectrum (Kubelka-Munk) of dehydroabietohydroxamic acid (**2a**).

Part B: Computational results

1. Computational details

2. Initial stability analysis (Amide vs. ester)

Figure S16. Optimized structures and relative energies for the stability analysis of initial species: (a) acido-amida (neutral), (b) acido-ester (neutral).

Table S1. M06-2X/6-31G(d,p) Cartesian coordinates and electronic energy (E, au) for acido-amida (neutral) in DMF.

Table S2. M06-2X/6-31G(d,p) Cartesian coordinates and electronic energy (E, au) for acido-ester (neutral) in DMF.

3. Transition states and intermediates (N-attack pathway)

Figure S17. Optimized structures and relative energies for the N-attack pathway: (a) Reactant complex (RC), (b) Transition state (TS), and (c) Product complex (PC).

Table S3. M06-2X/6-31G(d,p) Cartesian coordinates and electronic energy (E, au) for the reactant complex (RC) of the N-attack pathway.

Table S4. M06-2X/6-31G(d,p) Cartesian coordinates and electronic energy (E, au) for the transition state (TS) of the N-attack pathway.

Table S5. M06-2X/6-31G(d,p) Cartesian coordinates and electronic energy (E, au) for the product complex (PC) of the N-attack pathway.

4. Transition states and intermediates (O-attack pathway)

Figure S18. Optimized structures and relative energies for the O-attack pathway: (a) Reactant complex (RC), (b) Transition state (TS), and (c) Product complex (PC).

Table S6. M06-2X/6-31G(d,p) Cartesian coordinates and electronic energy (E, au) for the reactant complex (RC) of the O-attack pathway.

Table S7. M06-2X/6-31G(d,p) Cartesian coordinates and electronic energy (E, au) for the transition state (TS) of the O-attack pathway.

Table S8. M06-2X/6-31G(d,p) Cartesian coordinates and electronic energy (E, au) for the product complex (PC) of the O-attack pathway.

1. Computational details

All quantum-chemical calculations were performed using the Gaussian 09 (Revision D.01) software package. Electronic structures and geometries were optimized using the M06-2X hybrid meta-exchange-correlation functional and the 6-31G(d,p) basis set. Solvent effects were incorporated implicitly through the Polarizable Continuum Model (PCM) using N,N-dimethylformamide (DMF, ε = 37.22) as the solvent to match experimental conditions. To ensure a high-quality representation and analysis of the calculated stationary points, all molecular structures and three-dimensional representations were visualized and rendered using the CYLview software package.

2. Initial stability analysis (Amide vs. ester)

The relative stability of the potential initial species and intermediates was evaluated to understand the preferred reaction pathway. The values shown below correspond to relative electronic energies (ΔE, kcal/mol) calculated at the M06-2X/6-31G(d,p) level in DMF.

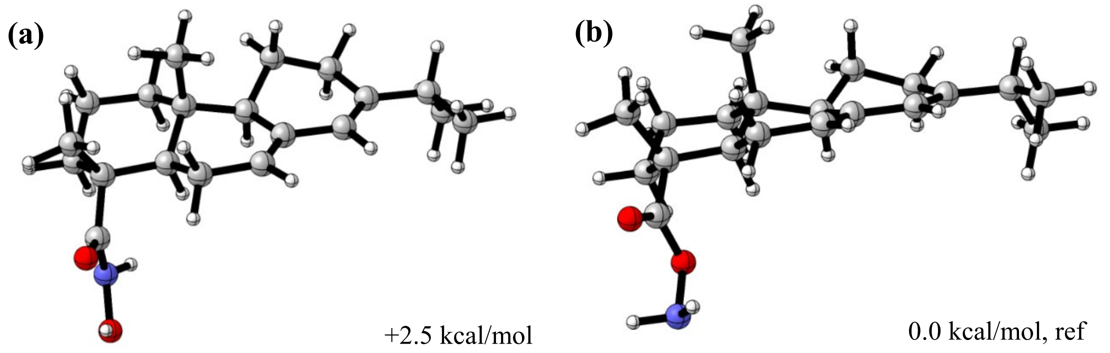

Figure S16. Optimized structures and relative energies for the stability analysis of initial species: (a) acido-amida (neutral), (b) acido-ester (neutral).

The results indicate that in their neutral form, the ester structure is slightly more stable than the amide (+2.5 kcal/mol), suggesting that the ester would predominate under thermodynamic control.

Table S1. M06-2X/6-31G(d,p) Cartesian coordinates and electronic energy (E, au) for acido-amida (neutral) in DMF.

HF=-985.5641563

Center Number	Atomic Number	Atomic Type	Coordinates (Angstroms) X	Y	Z
1	6	0	2.872061	-0.403474	0.127379
2	6	0	1.362839	-0.031055	0.231886
3	6	0	0.357254	-1.215715	0.159824
4	6	0	0.678269	-2.033567	-1.111063
5	6	0	2.143552	-2.469735	-1.181913
6	6	0	3.088926	-1.270679	-1.133461
7	6	0	1.033025	0.896610	1.402100
8	6	0	-0.292976	1.596832	1.123227
9	6	0	-1.352667	0.670132	0.563074
10	6	0	-1.057333	-0.615665	0.084418
11	6	0	-2.671481	1.142203	0.502309
12	6	0	-3.717239	0.383672	-0.012291
13	6	0	-3.416524	-0.903262	-0.476490
14	6	0	-2.116781	-1.385865	-0.421833
15	6	0	-5.132058	0.927732	-0.067391
16	6	0	-6.091649	0.074654	0.768287
17	6	0	-5.628550	1.034594	-1.512889
18	6	0	0.362180	-2.162171	1.378637
19	6	0	3.433457	-1.108291	1.367704
20	6	0	3.650322	0.923539	-0.004021
21	1	0	1.145170	0.543608	-0.682638
22	8	0	4.410131	1.349938	0.850128
23	7	0	3.478042	1.597323	-1.183832
24	8	0	3.884403	2.924223	-1.227299
25	1	0	0.039197	-2.921211	-1.158002
26	1	0	0.444096	-1.420892	-1.992639
27	1	0	2.370373	-3.170665	-0.371031
28	1	0	2.309975	-3.018784	-2.114771
29	1	0	2.910301	-0.670572	-2.033143
30	1	0	4.135060	-1.596833	-1.172057
31	1	0	0.975268	0.329435	2.336789
32	1	0	1.820859	1.644937	1.540869
33	1	0	-0.677885	2.082003	2.026581
34	1	0	-0.121726	2.400801	0.394377
35	1	0	-2.879296	2.144168	0.876304
36	1	0	-4.204863	-1.533466	-0.881534
37	1	0	-1.922645	-2.392721	-0.779914
38	1	0	-5.110164	1.936933	0.361637
39	1	0	-5.757599	0.003957	1.807777
40	1	0	-7.097353	0.506946	0.759931
41	1	0	-6.160881	-0.941319	0.364616
42	1	0	-4.963829	1.660352	-2.116021
43	1	0	-5.678548	0.044917	-1.979945
44	1	0	-6.632120	1.470839	-1.544206
45	1	0	1.257422	-2.782576	1.432128
46	1	0	0.263317	-1.620670	2.323451
47	1	0	-0.495307	-2.838773	1.301050
48	1	0	3.123779	-2.151506	1.412902
49	1	0	4.525270	-1.083203	1.338245
50	1	0	3.120566	-0.613576	2.290827
51	1	0	2.688643	1.430964	-1.794151
52	1	0	4.842599	2.879430	-1.368201

Table S2. M06-2X/6-31G(d,p) Cartesian coordinates and electronic energy (E, au) for acido-ester (neutral) in DMF.

HF=-985.5681072

Center Number	Atomic Number	Atomic Type	Coordinates (Angstroms) X	Y	Z
1	6	0	2.866216	-0.399128	0.145205
2	6	0	1.361020	-0.022808	0.226332
3	6	0	0.359052	-1.208217	0.160762
4	6	0	0.683762	-2.025041	-1.110612
5	6	0	2.151619	-2.454335	-1.181355
6	6	0	3.098047	-1.256496	-1.121604
7	6	0	1.026767	0.922088	1.380322
8	6	0	-0.292721	1.623268	1.076560
9	6	0	-1.352450	0.684047	0.537576
10	6	0	-1.056229	-0.610087	0.082701
11	6	0	-2.672980	1.151354	0.474602
12	6	0	-3.719302	0.379916	-0.019106
13	6	0	-3.417553	-0.915172	-0.459593
14	6	0	-2.116246	-1.392794	-0.402833
15	6	0	-5.136815	0.917034	-0.072896
16	6	0	-6.079727	0.088502	0.805598
17	6	0	-5.655638	0.973322	-1.513295
18	6	0	0.366132	-2.153784	1.380438
19	6	0	3.427460	-1.088468	1.392601
20	6	0	3.656914	0.899838	-0.028911
21	1	0	1.166535	0.538659	-0.698665
22	8	0	4.602267	1.255690	0.632381
23	8	0	3.183334	1.647486	-1.056698
24	7	0	3.887167	2.862125	-1.298128
25	1	0	0.049646	-2.916350	-1.157248
26	1	0	0.446481	-1.413286	-1.991614
27	1	0	2.380004	-3.158711	-0.373625
28	1	0	2.322632	-2.996947	-2.117031
29	1	0	2.927464	-0.630385	-2.005055
30	1	0	4.143307	-1.586077	-1.151231
31	1	0	0.959640	0.371930	2.324678
32	1	0	1.815637	1.672606	1.514645
33	1	0	-0.680106	2.135262	1.963773
34	1	0	-0.111716	2.404455	0.325667
35	1	0	-2.881748	2.159653	0.830492
36	1	0	-4.206523	-1.555570	-0.846975
37	1	0	-1.920549	-2.406228	-0.741360
38	1	0	-5.112993	1.940033	0.321962
39	1	0	-5.730224	0.055402	1.841881
40	1	0	-7.087917	0.515004	0.797118
41	1	0	-6.148633	-0.941133	0.437970
42	1	0	-5.002202	1.579895	-2.147517
43	1	0	-5.710291	-0.031652	-1.945820
44	1	0	-6.660850	1.405810	-1.543667
45	1	0	1.246633	-2.796592	1.413786
46	1	0	0.302760	-1.609335	2.326876
47	1	0	-0.508430	-2.809996	1.323471
48	1	0	3.079407	-2.116815	1.473792
49	1	0	4.518470	-1.106425	1.341639
50	1	0	3.147323	-0.555846	2.306180
51	1	0	3.921524	3.334665	-0.391536
52	1	0	4.855776	2.579446	-1.466104

3. Transition states and intermediates (N-attack pathway)

The N-attack pathway involves the nucleophilic attack of the hydroxylamine nitrogen on the carbonyl carbon of the activated abietic acid.

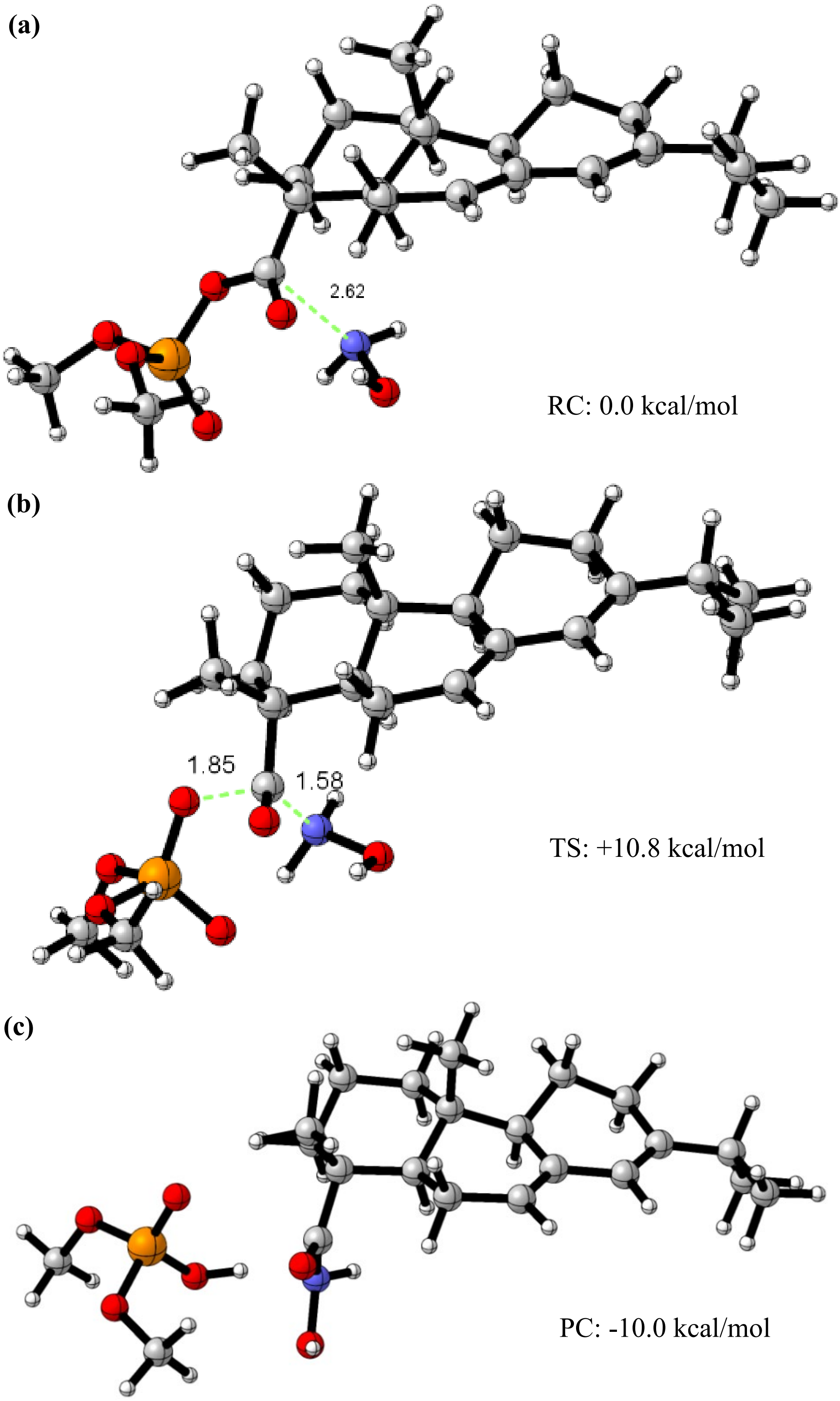

Figure S17. Optimized structures and relative energies for the N-attack pathway: (a) Reactant complex (RC), (b) Transition state (TS), and (c) Product complex (PC).

Table S3. M06-2X/6-31G(d,p) Cartesian coordinates and electronic energy (E, au) for the reactant complex (RC) of the N-attack pathway.

HF=-1708.14486451

Center Number	Atomic Number	Atomic Type	Coordinates (Angstroms) X	Y	Z
1	6	0	-1.317234	2.159207	-0.688646
2	6	0	-1.103042	1.123450	0.437481
3	6	0	0.350506	0.540665	0.421688
4	6	0	1.498485	1.575559	0.193646
5	6	0	1.133000	2.533292	-0.952527
6	6	0	-0.215832	3.208912	-0.741699
7	1	0	-1.342954	1.632232	-1.648014
8	1	0	-2.295623	2.629032	-0.554872
9	1	0	1.915815	3.289742	-1.067957
10	1	0	1.095472	1.963441	-1.892591
11	1	0	-0.204034	3.813920	0.173123
12	1	0	-0.421703	3.901423	-1.564563
13	1	0	0.373943	-0.121172	-0.457647
14	6	0	1.819001	2.388917	1.462757
15	1	0	2.280862	1.759310	2.228347
16	1	0	2.522917	3.192671	1.225765
17	1	0	0.935573	2.853064	1.899438
18	6	0	-1.552297	1.736109	1.782642
19	1	0	-2.615521	1.980152	1.735226
20	1	0	-1.397162	1.050492	2.616865
21	1	0	-1.017062	2.663673	1.991157
22	6	0	2.753731	0.761090	-0.218899
23	1	0	2.538857	0.322118	-1.208743
24	6	0	0.697346	-0.330273	1.639795
25	1	0	-0.005783	-1.161589	1.720647
26	1	0	0.587463	0.247420	2.567977
27	6	0	3.038903	-0.389651	0.732232
28	6	0	2.098534	-0.864784	1.562983
29	6	0	4.030890	1.593000	-0.385202
30	1	0	3.859000	2.438184	-1.059006
31	1	0	4.335498	1.999598	0.585775
32	6	0	4.387799	-0.969201	0.701729
33	6	0	5.161686	0.722550	-0.927725
34	6	0	5.384399	-0.500556	-0.067688
35	1	0	4.929436	0.421928	-1.959263
36	1	0	6.093055	1.299724	-0.983638
37	1	0	4.553362	-1.830914	1.345307
38	1	0	2.353061	-1.687192	2.229492
39	6	0	6.759285	-1.130863	-0.153021
40	1	0	7.486444	-0.319039	-0.001623
41	6	0	6.987001	-1.688239	-1.567538
42	1	0	6.264952	-2.484885	-1.773285
43	1	0	6.868224	-0.917172	-2.331023
44	1	0	7.993733	-2.105752	-1.660392
45	6	0	7.031379	-2.206706	0.893915
46	1	0	8.067045	-2.549841	0.816461
47	1	0	6.871259	-1.832021	1.907586
48	1	0	6.381943	-3.074894	0.744072
49	6	0	-2.098772	-0.015634	0.261440
50	8	0	-1.985357	-1.164353	0.626251
51	7	0	-1.469226	-0.801208	-2.157525
52	8	0	-1.100222	-2.141928	-1.849379
53	1	0	-1.228109	-2.176065	-0.890242
54	1	0	-0.745962	-0.471530	-2.789876
55	1	0	-2.335191	-0.869847	-2.691982
56	8	0	-3.309946	0.441331	-0.210865
57	15	0	-4.559755	-0.592389	-0.301411
58	8	0	-4.451709	-1.648856	-1.321275
59	8	0	-4.827423	-1.082110	1.186114
60	8	0	-5.669804	0.527907	-0.510231
61	6	0	-4.663027	-2.467565	1.545376
62	1	0	-5.173532	-2.590166	2.499073
63	1	0	-5.117813	-3.108349	0.786220
64	1	0	-3.600277	-2.694735	1.644889
65	6	0	-7.049455	0.127916	-0.399158
66	1	0	-7.256118	-0.196020	0.620959
67	1	0	-7.640073	1.003915	-0.642913
68	1	0	-7.268134	-0.675662	-1.103315

Table S4. M06-2X/6-31G(d,p) Cartesian coordinates and electronic energy (E, au) for the transition state (TS) of the N-attack pathway.

HF=-1708.1284705; 1 imaginary frequencies=-258.45

Center Number	Atomic Number	Atomic Type	Coordinates (Angstroms) X	Y	Z
1	6	0	-1.304827	1.982733	-0.496624
2	6	0	-1.027755	0.926864	0.591517
3	6	0	0.463667	0.420480	0.495378
4	6	0	1.563754	1.511823	0.299591
5	6	0	1.125588	2.512761	-0.781470
6	6	0	-0.258188	3.091786	-0.521041
7	1	0	-1.317888	1.521710	-1.494579
8	1	0	-2.303149	2.392318	-0.338310
9	1	0	1.862718	3.318630	-0.859492
10	1	0	1.110593	2.000303	-1.754970
11	1	0	-0.267318	3.651323	0.422046
12	1	0	-0.517893	3.809591	-1.305413
13	1	0	0.517781	-0.175154	-0.430632
14	6	0	1.890910	2.253270	1.606794
15	1	0	2.409220	1.596794	2.312181
16	1	0	2.544816	3.106450	1.402331
17	1	0	0.999859	2.638664	2.103426
18	6	0	-1.438525	1.494030	1.959861
19	1	0	-2.524003	1.622654	1.969150
20	1	0	-1.169951	0.825962	2.778780
21	1	0	-0.986725	2.469994	2.137951
22	6	0	2.832575	0.766315	-0.202453
23	1	0	2.606806	0.405334	-1.220286
24	6	0	0.875085	-0.524565	1.632094
25	1	0	0.198281	-1.381052	1.683558
26	1	0	0.778676	-0.017329	2.602311
27	6	0	3.174478	-0.449249	0.637053
28	6	0	2.282834	-1.013611	1.463405
29	6	0	4.084976	1.646040	-0.322760
30	1	0	3.873306	2.546357	-0.907211
31	1	0	4.403526	1.968669	0.675392
32	6	0	4.525795	-1.004112	0.491755
33	6	0	5.224913	0.862765	-0.972380
34	6	0	5.485406	-0.448806	-0.266256
35	1	0	4.987789	0.675317	-2.029453
36	1	0	6.143947	1.462579	-0.972928
37	1	0	4.718246	-1.929877	1.029472
38	1	0	2.580660	-1.881792	2.050074
39	6	0	6.867013	-1.042306	-0.446806
40	1	0	7.582408	-0.295730	-0.068735
41	6	0	7.170578	-1.246229	-1.939906
42	1	0	6.458542	-1.955784	-2.375020
43	1	0	7.106592	-0.311314	-2.502248
44	1	0	8.178359	-1.649977	-2.072641
45	6	0	7.093153	-2.346616	0.312942
46	1	0	8.117030	-2.697680	0.159037
47	1	0	6.934982	-2.222769	1.387853
48	1	0	6.412272	-3.126509	-0.044531
49	6	0	-1.895362	-0.334903	0.420486
50	8	0	-2.067984	-1.171598	1.290982
51	7	0	-1.743954	-1.049245	-0.983894
52	8	0	-0.935484	-2.177954	-0.824126
53	1	0	-1.228126	-2.512244	0.048413
54	1	0	-1.301223	-0.470970	-1.700441
55	1	0	-2.721554	-1.368016	-1.278312
56	8	0	-3.505698	0.453820	-0.017833
57	15	0	-4.697917	-0.413316	-0.454981
58	8	0	-4.292146	-1.657677	-1.202710
59	8	0	-5.661080	-0.752743	0.780631
60	8	0	-5.636137	0.567997	-1.296114
61	6	0	-5.231956	-1.767689	1.700421
62	1	0	-6.008924	-1.848442	2.458925
63	1	0	-5.116692	-2.721394	1.180217
64	1	0	-4.281620	-1.482868	2.159183
65	6	0	-6.827854	0.013167	-1.873482
66	1	0	-7.541479	-0.242115	-1.086844
67	1	0	-7.247800	0.780385	-2.521652
68	1	0	-6.584946	-0.876005	-2.459982

Table S5. M06-2X/6-31G(d,p) Cartesian coordinates and electronic energy (E, au) for the product complex (PC) of the N-attack pathway.

HF=-1708.16081229

Center Number	Atomic Number	Atomic Type	Coordinates (Angstroms) X	Y	Z
1	6	0	-1.240366	1.520500	-0.216038
2	6	0	-0.808478	0.427635	0.787096
3	6	0	0.709464	0.081081	0.569135
4	6	0	1.688600	1.289272	0.475534
5	6	0	1.106649	2.356121	-0.471072
6	6	0	-0.331702	2.744632	-0.142207
7	1	0	-1.187941	1.139009	-1.244376
8	1	0	-2.285454	1.776941	-0.019757
9	1	0	1.745601	3.245232	-0.459512
10	1	0	1.129368	1.962095	-1.497819
11	1	0	-0.388481	3.213574	0.846845
12	1	0	-0.683113	3.495339	-0.857193
13	1	0	0.761513	-0.379175	-0.431814
14	6	0	2.000857	1.907269	1.847753
15	1	0	2.579116	1.218505	2.470809
16	1	0	2.592426	2.818597	1.720835
17	1	0	1.100022	2.179905	2.397326
18	6	0	-1.199572	0.852094	2.211507
19	1	0	-2.289892	0.867449	2.273762
20	1	0	-0.823863	0.160076	2.965787
21	1	0	-0.833878	1.851817	2.442105
22	6	0	2.996281	0.729549	-0.151501
23	1	0	2.757230	0.459413	-1.194068
24	6	0	1.236323	-0.967618	1.554085
25	1	0	0.638793	-1.884667	1.489412
26	1	0	1.118767	-0.614890	2.588082
27	6	0	3.483875	-0.535952	0.532741
28	6	0	2.679295	-1.287957	1.297952
29	6	0	4.152879	1.735534	-0.222057
30	1	0	3.829542	2.663080	-0.703927
31	1	0	4.483270	1.988354	0.792213
32	6	0	4.879828	-0.927063	0.299635
33	6	0	5.330870	1.136365	-0.987269
34	6	0	5.751615	-0.199066	-0.416562
35	1	0	5.060086	1.019873	-2.046633
36	1	0	6.185963	1.823721	-0.969270
37	1	0	5.186528	-1.875489	0.735906
38	1	0	3.081919	-2.184734	1.767065
39	6	0	7.175119	-0.628033	-0.701572
40	1	0	7.825324	0.186141	-0.345956
41	6	0	7.398489	-0.756632	-2.216910
42	1	0	6.751114	-1.539356	-2.626631
43	1	0	7.178464	0.176470	-2.741532
44	1	0	8.437408	-1.025559	-2.428723
45	6	0	7.599307	-1.912472	0.004925
46	1	0	8.651042	-2.125587	-0.205469
47	1	0	7.476831	-1.835502	1.089099
48	1	0	7.008544	-2.766035	-0.345208
49	6	0	-1.564708	-0.872241	0.522191
50	8	0	-1.952596	-1.644424	1.380112
51	7	0	-1.812813	-1.203617	-0.821776
52	8	0	-2.238710	-2.524721	-1.000653
53	1	0	-2.165076	-2.911122	-0.105622
54	1	0	-1.043725	-1.013066	-1.462064
55	1	0	-3.348065	-0.446069	-1.404290
56	8	0	-4.200280	0.639093	0.838378
57	15	0	-5.073233	0.131006	-0.241255
58	8	0	-4.308701	-0.271325	-1.576154
59	8	0	-5.962237	-1.145386	0.129682
60	8	0	-6.179628	1.171305	-0.695721
61	6	0	-5.257372	-2.378709	0.359711
62	1	0	-5.998585	-3.105244	0.686922
63	1	0	-4.782255	-2.713538	-0.565746
64	1	0	-4.495472	-2.242089	1.133524
65	6	0	-7.085837	0.823334	-1.757971
66	1	0	-7.734060	0.004246	-1.439814
67	1	0	-7.677490	1.713291	-1.962955
68	1	0	-6.524620	0.537038	-2.649851

4. Transition states and intermediates (O-attack pathway)

The O-attack pathway involves the nucleophilic attack of the hydroxylamine oxygen on the carbonyl carbon of the activated abietic acid.

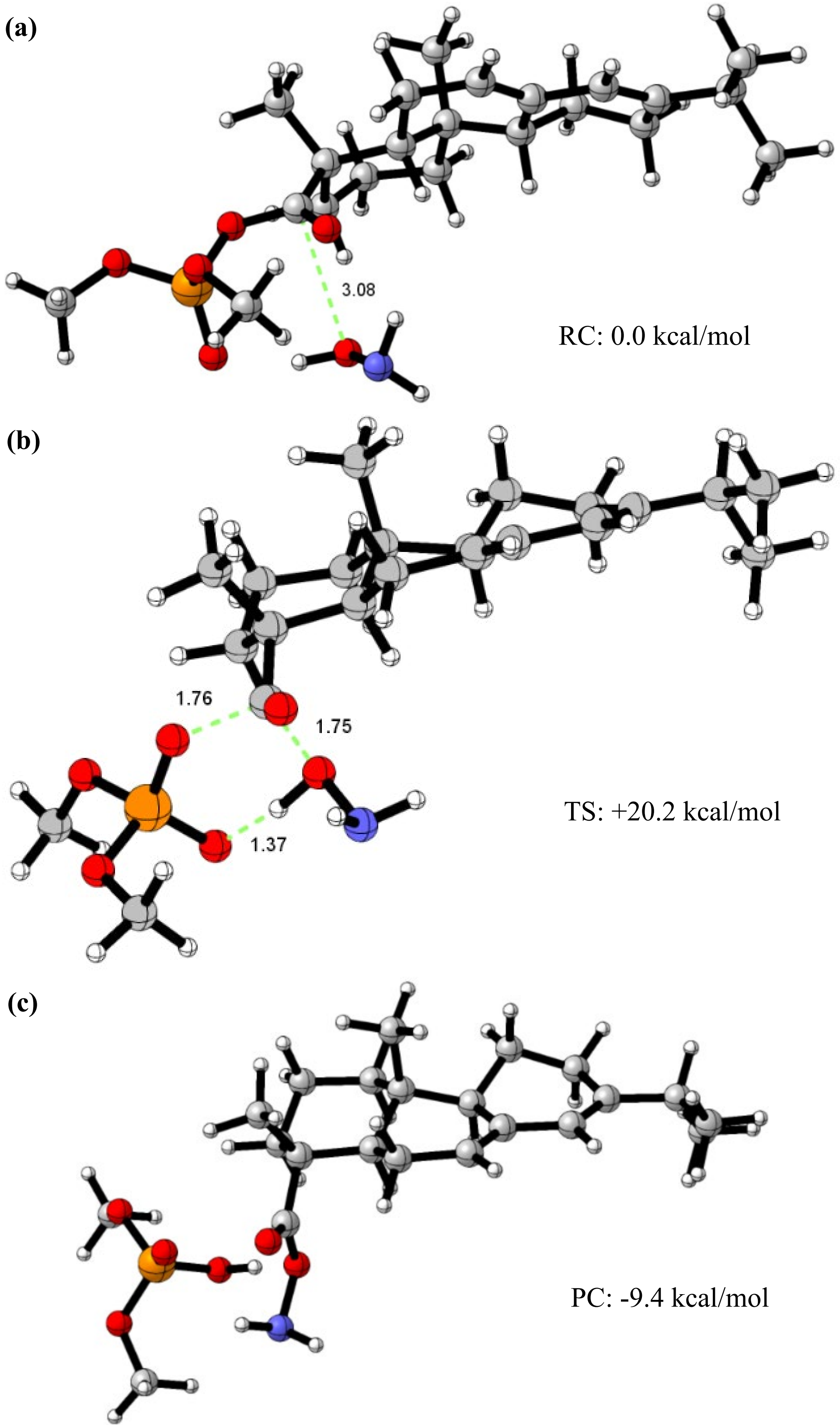

Figure S18. Optimized structures and relative energies for the O-attack pathway: (a) Reactant complex (RC), (b) Transition state (TS), and (c) Product complex (PC).

Table S6. M06-2X/6-31G(d,p) Cartesian coordinates and electronic energy (E, au) for the reactant complex (RC) of the O-attack pathway.

HF=-1708.14969929

Center Number	Atomic Number	Atomic Type	Coordinates (Angstroms) X	Y	Z
1	6	0	-1.299048	2.038788	-0.984659
2	6	0	-1.062251	1.317925	0.368944
3	6	0	0.384811	0.751730	0.447165
4	6	0	1.541465	1.680976	-0.029735
5	6	0	1.152978	2.358940	-1.354517
6	6	0	-0.202755	3.054622	-1.284629
7	1	0	-1.317938	1.287091	-1.784242
8	1	0	-2.284486	2.514720	-0.962634
9	1	0	1.928495	3.076070	-1.644180
10	1	0	1.110908	1.596346	-2.145849
11	1	0	-0.188701	3.845858	-0.525233
12	1	0	-0.420452	3.545714	-2.238358
13	1	0	0.389723	-0.086821	-0.265476
14	6	0	1.923783	2.748310	1.009323
15	1	0	2.372436	2.295235	1.898130
16	1	0	2.656204	3.440714	0.583757
17	1	0	1.069857	3.345105	1.332558
18	6	0	-1.476468	2.238539	1.533469
19	1	0	-2.521228	2.533867	1.417430
20	1	0	-1.367643	1.747734	2.503372
21	1	0	-0.874463	3.147035	1.542969
22	6	0	2.756996	0.743696	-0.274635
23	1	0	2.502907	0.118720	-1.147211
24	6	0	0.720216	0.159726	1.819613
25	1	0	-0.021955	-0.597954	2.092388
26	1	0	0.661467	0.935290	2.596639
27	6	0	3.016723	-0.198553	0.887952
28	6	0	2.091889	-0.447638	1.826934
29	6	0	4.065850	1.464200	-0.624944
30	1	0	3.912283	2.169255	-1.447747
31	1	0	4.410977	2.040293	0.241706
32	6	0	4.328487	-0.856832	0.931795
33	6	0	5.145318	0.450948	-1.002473
34	6	0	5.319106	-0.612243	0.058662
35	1	0	4.888981	-0.019788	-1.962485
36	1	0	6.104412	0.959228	-1.164418
37	1	0	4.460841	-1.597374	1.717681
38	1	0	2.336575	-1.126363	2.643137
39	6	0	6.654934	-1.325143	0.076407
40	1	0	7.423343	-0.551756	0.229669
41	6	0	6.931251	-1.974638	-1.289351
42	1	0	6.168976	-2.730619	-1.507121
43	1	0	6.925211	-1.241774	-2.100266
44	1	0	7.908468	-2.466128	-1.287194
45	6	0	6.796137	-2.365417	1.184257
46	1	0	7.791283	-2.816983	1.149882
47	1	0	6.657331	-1.924840	2.175389
48	1	0	6.058500	-3.165296	1.059776
49	6	0	-2.012219	0.127712	0.362598
50	8	0	-1.734388	-1.042247	0.387330
51	8	0	-3.327919	0.534187	0.300025
52	15	0	-4.525766	-0.528138	0.004916
53	8	0	-4.629405	-0.929032	-1.415893
54	8	0	-4.402769	-1.715646	1.036594
55	8	0	-5.697395	0.354433	0.593698
56	6	0	-3.872141	-3.001176	0.638088
57	1	0	-3.131611	-3.281903	1.385123
58	1	0	-4.699540	-3.710548	0.632661
59	1	0	-3.403715	-2.942127	-0.345785
60	6	0	-7.023747	-0.212774	0.576833
61	1	0	-7.056675	-1.093623	1.220698
62	1	0	-7.688442	0.557364	0.960711
63	1	0	-7.305865	-0.476712	-0.444126
64	8	0	-2.102908	-0.861575	-2.553228
65	1	0	-3.055468	-0.838658	-2.356063
66	7	0	-1.761296	-2.213039	-2.245826
67	1	0	-1.169551	-2.500947	-3.023517
68	1	0	-1.145501	-2.143214	-1.435520

Table S7. M06-2X/6-31G(d,p) Cartesian coordinates and electronic energy (E, au) for the transition state (TS) of the O-attack pathway.

HF=-1708.1174978; 1 imaginary frequencies=-357.24

Center Number	Atomic Number	Atomic Type	Coordinates (Angstroms) X	Y	Z
1	6	0	-1.578166	1.553816	-0.138016
2	6	0	-1.091781	0.580390	0.951238
3	6	0	0.423124	0.211399	0.744373
4	6	0	1.385443	1.389562	0.389544
5	6	0	0.749967	2.281624	-0.689176
6	6	0	-0.648785	2.750120	-0.310591
7	1	0	-1.640040	1.032492	-1.101388
8	1	0	-2.586786	1.889704	0.115635
9	1	0	1.395275	3.144775	-0.882554
10	1	0	0.685019	1.713389	-1.628580
11	1	0	-0.616477	3.350747	0.606439
12	1	0	-1.050565	3.403575	-1.091205
13	1	0	0.434036	-0.426971	-0.150592
14	6	0	1.762774	2.238448	1.615826
15	1	0	2.410223	1.680485	2.298417
16	1	0	2.306030	3.133650	1.298590
17	1	0	0.891143	2.571953	2.180231
18	6	0	-1.440051	1.146417	2.340584
19	1	0	-2.526306	1.224528	2.429382
20	1	0	-1.076844	0.504888	3.145213
21	1	0	-1.021986	2.144594	2.472949
22	6	0	2.667682	0.734087	-0.196035
23	1	0	2.380942	0.283720	-1.161470
24	6	0	1.022981	-0.613859	1.890446
25	1	0	0.441029	-1.525528	2.049376
26	1	0	0.966125	-0.055399	2.835679
27	6	0	3.205645	-0.384644	0.678345
28	6	0	2.452756	-0.975626	1.617020
29	6	0	3.809452	1.714903	-0.497267
30	1	0	3.456169	2.543437	-1.118199
31	1	0	4.184763	2.141904	0.440009
32	6	0	4.590849	-0.811991	0.446501
33	6	0	4.957964	0.994368	-1.201522
34	6	0	5.420518	-0.220191	-0.427779
35	1	0	4.640055	0.694171	-2.210318
36	1	0	5.804927	1.677448	-1.343937
37	1	0	4.926019	-1.668906	1.027117
38	1	0	2.891000	-1.767161	2.223920
39	6	0	6.834461	-0.687588	-0.698780
40	1	0	7.491668	0.174286	-0.505238
41	6	0	6.998830	-1.058516	-2.181045
42	1	0	6.342302	-1.898061	-2.433604
43	1	0	6.751855	-0.222277	-2.840359
44	1	0	8.030550	-1.357355	-2.387953
45	6	0	7.296876	-1.841241	0.186934
46	1	0	8.341360	-2.082711	-0.027904
47	1	0	7.215856	-1.593085	1.248898
48	1	0	6.700205	-2.740629	-0.000225
49	6	0	-1.856103	-0.745456	0.891397
50	8	0	-1.806465	-1.682951	1.620750
51	8	0	-3.546469	-0.264861	0.759390
52	15	0	-4.459090	-0.208116	-0.473922
53	8	0	-3.777086	-0.646776	-1.756176
54	8	0	-5.772844	-1.070391	-0.189716
55	8	0	-5.049502	1.263032	-0.555840
56	6	0	-5.584961	-2.486805	-0.032312
57	1	0	-4.906248	-2.681011	0.803191
58	1	0	-6.564294	-2.911609	0.178341
59	1	0	-5.179247	-2.916638	-0.951179
60	6	0	-5.916797	1.588560	-1.657920
61	1	0	-6.850948	1.029614	-1.573699
62	1	0	-6.113574	2.656440	-1.589610
63	1	0	-5.422773	1.358635	-2.604226
64	8	0	-1.664286	-1.297597	-0.762160
65	1	0	-2.539174	-1.034489	-1.323702
66	7	0	-1.578312	-2.710621	-0.834097
67	1	0	-0.574486	-2.890428	-0.815011
68	1	0	-1.925110	-3.036401	0.074498

Table S8. M06-2X/6-31G(d,p) Cartesian coordinates and electronic energy (E, au) for the product complex (PC) of the O-attack pathway.

HF=-1708.16469848

Center Number	Atomic Number	Atomic Type	Coordinates (Angstroms) X	Y	Z
1	6	0	-1.397565	1.301529	0.162109
2	6	0	-0.826142	0.286844	1.172939
3	6	0	0.674338	-0.009707	0.819414
4	6	0	1.585271	1.228540	0.571775
5	6	0	0.868057	2.225647	-0.357892
6	6	0	-0.548954	2.568964	0.095686
7	1	0	-1.402573	0.859880	-0.840281
8	1	0	-2.436062	1.531720	0.427847
9	1	0	1.462914	3.141085	-0.445237
10	1	0	0.809768	1.787843	-1.365055
11	1	0	-0.528973	3.079719	1.065252
12	1	0	-1.002361	3.269826	-0.612147
13	1	0	0.628035	-0.522468	-0.155140
14	6	0	1.998668	1.926322	1.877681
15	1	0	2.666482	1.294776	2.470935
16	1	0	2.529908	2.856456	1.655598
17	1	0	1.142682	2.185863	2.500730
18	6	0	-1.107246	0.767241	2.605735
19	1	0	-2.187609	0.745791	2.774497
20	1	0	-0.644211	0.122792	3.353582
21	1	0	-0.768137	1.789419	2.764290
22	6	0	2.851586	0.690391	-0.151977
23	1	0	2.526599	0.356648	-1.151774
24	6	0	1.340548	-0.981525	1.798786
25	1	0	0.782858	-1.924379	1.838056
26	1	0	1.308591	-0.579297	2.821319
27	6	0	3.458440	-0.515287	0.542790
28	6	0	2.765102	-1.257079	1.418908
29	6	0	3.949864	1.737226	-0.382101
30	1	0	3.540413	2.623074	-0.876749
31	1	0	4.361248	2.058252	0.581826
32	6	0	4.843528	-0.860443	0.199716
33	6	0	5.080906	1.147563	-1.223176
34	6	0	5.614261	-0.134967	-0.626532
35	1	0	4.721477	0.961608	-2.245445
36	1	0	5.899419	1.871927	-1.319261
37	1	0	5.230748	-1.770902	0.651894
38	1	0	3.251718	-2.109160	1.891613
39	6	0	7.028043	-0.514695	-1.012281
40	1	0	7.665111	0.343047	-0.747484
41	6	0	7.134469	-0.704434	-2.533514
42	1	0	6.492091	-1.531021	-2.854792
43	1	0	6.832935	0.193415	-3.078750
44	1	0	8.163969	-0.941357	-2.817395
45	6	0	7.568504	-1.744664	-0.288785
46	1	0	8.609312	-1.920397	-0.573862
47	1	0	7.527877	-1.623798	0.797161
48	1	0	6.993518	-2.638137	-0.554118
49	6	0	-1.533249	-1.059970	1.055433
50	8	0	-1.790722	-1.804482	1.966043
51	8	0	-4.307475	-0.546537	0.853998
52	15	0	-4.859237	-0.049691	-0.426463
53	8	0	-3.837207	-0.094466	-1.651557
54	8	0	-6.156317	-0.792313	-0.988726
55	8	0	-5.357952	1.452724	-0.338079
56	6	0	-6.021466	-2.176758	-1.350461
57	1	0	-5.814206	-2.771623	-0.460299
58	1	0	-6.971065	-2.479749	-1.786836
59	1	0	-5.213540	-2.298319	-2.075953
60	6	0	-5.864440	2.106484	-1.516715
61	1	0	-6.780515	1.614080	-1.848169
62	1	0	-6.073010	3.135792	-1.231375
63	1	0	-5.113022	2.083153	-2.308646
64	8	0	-1.813630	-1.401523	-0.240093
65	1	0	-2.988168	-0.490149	-1.373857
66	7	0	-2.566339	-2.605865	-0.390265
67	1	0	-1.879946	-3.352953	-0.264588
68	1	0	-3.160064	-2.636590	0.443476
